# Anti-IgLON5 antibodies cause progressive behavioral and neuropathological changes in mice

**DOI:** 10.1186/s12974-022-02520-z

**Published:** 2022-06-11

**Authors:** You Ni, Yifan Feng, Dingding Shen, Ming Chen, Xiaona Zhu, Qinming Zhou, Yining Gao, Jun Liu, Qi Zhang, Yuntian Shen, Lisheng Peng, Zike Zeng, Dou Yin, Ji Hu, Sheng Chen

**Affiliations:** 1 Department of Neurology & Institute of Neurology, Ruijin Hospital, Affiliated with Shanghai Jiao Tong University School of Medicine, Shanghai, 200025 China; 2 School of Life Science and Technology, ShanghaiTech University, Shanghai, 201210 China; 3 Co-Innovation Center of Neuroregeneration, Nantong University, Nantong, 226019 China; 4 Key Laboratory of Neuroregeneration of Jiangsu and Ministry of Education, Nantong University, Nantong, 226019 China; 5 Department of Neurology, The Third Affiliated Hospital of Sun Yat-Sen University, Guangzhou, 510630 China; 6 Shanghai Key Laboratory of Psychotic Disorders, Shanghai Mental Health Center, Shanghai, 200030 China

**Keywords:** Anti-IgLON5 disease, Animal model, Neuropathology, Pathogenesis

## Abstract

**Background:**

Anti-IgLON5 disease is a rare neurological disorder associated with autoantibodies against the neuronal cell adhesion protein, IgLON5. Cellular investigations with human IgLON5 antibodies have suggested an antibody-mediated pathogenesis, but whether human IgLON5 autoantibodies can induce disease symptoms in mice is yet to be shown. Moreover, the effects of anti-IgLON5 autoantibodies on neurons and the precise molecular mechanisms in vivo remain controversial.

**Methods:**

We investigated the effects of anti-IgLON5 antibodies in vivo and evaluated their long-term effects. We used two independent passive-transfer animal models and evaluated the effects of the antibodies on mouse behaviors at different time points from day 1 until day 30 after IgG infusion. A wide range of behaviors, including tests of locomotion, coordination, memory, anxiety, depression and social interactions were established. At termination, brain tissue was analyzed for human IgG, neuronal markers, glial markers, synaptic markers and RNA sequencing.

**Results:**

These experiments showed that patient’s anti-IgLON5 antibodies induced progressive and irreversible behavioral deficits in vivo. Notably, cognitive abnormality was supported by impaired average gamma power in the CA1 during novel object recognition testing. Accompanying brain tissue studies showed progressive increase of brain-bound human antibodies in the hippocampus of anti-IgLON5 IgG-injected mice, which persisted 30 days after the injection of patient’s antibodies was stopped. Microglial and astrocyte density was increased in the hippocampus of anti-IgLON5 IgG-injected mice at Day 30. Whole-cell voltage clamp recordings proved that anti-IgLON5 antibodies affected synaptic homeostasis. Further western blot investigation of synaptic proteins revealed a reduction of presynaptic (synaptophysin) and post-synaptic (PSD95 and NMDAR1) expression in anti-IgLON5 IgG-injected mice.

**Conclusions:**

Overall, our findings indicated an irreversible effect of anti-IgLON5 antibodies and supported the pathogenicity of these antibodies in vivo*.*

**Supplementary Information:**

The online version contains supplementary material available at 10.1186/s12974-022-02520-z.

## Introduction

Anti-IgLON5 disease is a recently described neurological disorder, characterized by sleep disorders, progressive gait instability, brainstem dysfunction, and cognitive impairment [1]. The biological hallmark of this disease is the presence of antibodies against IgLON5, a neuronal cell adhesion molecule whose function is not fully understood [2]. Whether the primary underlying pathophysiology of anti-IgLON5 disease is degenerative or autoimmune is unclear. Postmortem examinations have suggested a novel tauopathy with neuronal loss, predominantly involving the hypothalamus, tegmentum of the brainstem, hippocampus, and cerebellum [3]. However, a recent case report demonstrated that tauopathy was not always present in autopsy [4]. Despite the neurodegenerative features, a strong association of the disease with the HLA-DRB1*10:01-DQB1*05:01 haplotype and the presence of an antibody against a neuronal surface antigen supported an immune-mediated pathogenesis [5].

Cellular investigations of the potential pathogenic role of patients’ antibodies using cultured neurons showed that the antibodies cause damage to the neuronal cytoskeleton and lead to the internalization of surface IgLON5, which are irreversible in the long term after the removal of antibodies [6, 7]. Moreover, patient antibodies also result in dystrophic neurites and axonal swelling [7]. Collectively, these studies suggest antibody-mediated pathogenesis, providing a link between autoimmunity and neurodegeneration. However, whether patient-derived anti-IgLON5 antibodies can induce disease symptoms in vivo remains to be determined.

Passive immunization is ideal for investigating the pathogenicity of a given neuronal autoantibody, including behavioral changes and molecular synaptic abnormalities [8]. We therefore purified immunoglobulin G (IgG) autoantibodies from one patient with anti-IgLON5 disease and established two independent passive-transfer animal models. The short-term and long-term effects of anti-IgLON5 antibodies on mouse behaviors were examined at different time points from day 1 and day 30 after injections. The ventricle is a commonly chosen injection site in animal models of autoimmune encephalitis and increases parenchymal antibody binding [9, 10]. In addition, human antibodies can preferentially bind to the hippocampus during ventricular perfusion due to the close spatial relationship between the hippocampus and the ventricle. Our patient's PET/MR also indicated damaged metabolism in the temporal lobe, so we selected the hippocampus for local injection of antibodies. Our results thus identify a unique immune–neuronal interaction that may underlie characteristic disease symptoms. To our knowledge, this is the innovative study of anti-IgLON5 antibodies are causative for the disease symptoms in vivo.

## Materials and methods

### Animals

A total of 160 male C57BL/6 mice aged 7–8 weeks were purchased from Charles River (Beijing, China). Animal care and use strictly followed institutional guidelines and governmental regulations. The mice were housed in cages with 4–5 mice per cage, before surgery. After surgery, each mouse was housed in a separate cage based on the different behavioral experiments. They were under a 12-h light–dark cycle (light on from 9:00 p.m. to 9:00 a.m.) at 22–25 °C with free access to food and water. All in vivo and in vitro experiments were performed exactly as approved by the IACUC at Shanghai Jiaotong University and ShanghaiTech University.

### Human IgG purification and subclass analysis

IgG were purified using Protein A from a serum sample of a 74-year-old female patient with anti-IgLON5 disease and that of one healthy individual with matched age and sex (healthy control-IgG) as described previously [11]. The concentration of the patient’s purified IgG was 2.3 mg/mL while the one from healthy control was 1.8 mg/mL.

Antibody testing was done through indirect immunofluorescence analysis to test for anti-IgLON5 antibody (Jinyu Inspection Co., Ltd., Guangzhou, GD, China).

### Surgery

#### Stereotactic IgG injection

Daily stereotactic injections of purified human IgG or control IgG for 7 days into CA1 was performed in C57BL/6 male mice. The surgical approach was similar to that of Yuan et al., Li et al., Zeng et al. [12–14]. Briefly, C57BL/6 WT mice were adequately anesthetized using 1.5% of isoflurane/oxygen (w/v), and the head was fixed onto a stereotactic apparatus. The skull was exposed using a small midline incision; the stereotactic injection sites were marked, and holes were drilled with a dentist’s drill. Using a micro-syringe pump (Nanoject III #3-000-207, DRUMNOND), 2 μL purified human or control IgG (50 steps, 20 μL every 30 s) was injected daily into the dorsal side of both CA1 on the following coordinates: AP, − 1.4 mm; ML, ± 1.0 mm; DV, − 1.7 mm. The injector was kept still for an additional 10 min to allow the IgG to diffuse. Tissue damage in the target region was avoided by using very thin glass application pipettes, low injection volume, and slow injection speed. At the end of each day, the scalps of the mice were disinfected and cleaned to reduce infection, lidocaine ointment was applied locally to relieve pain, and a simple local dressing was performed to reduce exposure. After the injection on the seventh day, the skin was sutured.

#### Ventricular cannulas embedment

Cerebroventricular infusion of IgG was performed using cannulas. Cannulas were purchased from RWD Life Science Co., LTD (Shenzhen China). The procedure for implanting the cannula is the same as described above. Then cannulas were implanted into the ventricles (coordinates from Bregma: AP, − 0.46 mm; ML, ± 1.0 mm; DV, − 1.4 mm). The injection was started one week after the surgery. All injections were administered between 4–6 pm and continued for 10 days. Mice received 2 μl of purified IgG daily of either patient or healthy control IgG. The locations of the cannulas or microinjection in the ventricle and dorsal CA1 were defined by visualization of stained tissue under a microscope.

#### Behavioral testing

The same designated room was used for all behavioral studies and testing was performed during the dark phase (9:00–21:00). Three days before the experiment, all mice were handled for at least 5 min per day. Mice were placed in the room 2 h before tests to adapt to the test environment. The tasks were aimed to assess memory (novel object recognition and Y-maze), anxiety (elevated plus maze), locomotion (open field test), social behaviors (three-chamber social interaction test), depressive-like behaviors (tail suspension) and coordination (rotarod test). Change in body weight, water intake, food intake and anal temperature of mice after IgG infusion was monitored. A description of each task is provided in the Additional file 1.

#### Histology

The mice were deeply anesthetized through the abdominal cavity injection of tribromoethanol (20 mg/kg, i.p.), following saline perfusion through the heart. Most of the blood was drained and fixed with 4% paraformaldehyde (PFA). The stripped brain was placed in PFA overnight and then transferred to 30% sucrose at 4 °C for 24 to 48 h. The 40 μm thick coronal sections were obtained with a cryostat microtome (Leica CM3050S). Sections were blocked by pre-application of blocking buffers containing 5% bovine serum albumin (BSA) and 0.3% Triton X-100 in 1xPBS. The blocking buffers are used for subsequent primary antibody incubation. The primary antibodies used were rabbit polyclonal anti-NeuN (1:500, #26975-1-AP, Proteintech), anti-glial fibrillary acidic protein (GFAP) (Proteintech, #16828-1-AP, 1:100) and anti-IBA1 (Wako, #019-19741, 1:500). The secondary antibody used were AlexaFluor 488 goat anti-human IgG (H + L) (1:1000; #A-11013, Thermo Fisher) and AlexaFluor 488 donkey anti-rabbit IgG (1:1000; Jackson ImmunoResearch). The primary antibody was incubated at 4 °C for 48 h, and the secondary antibody was incubated at room temperature for 2 h. The sections were washed with 1xPBS buffer 3 times between changes in antibody incubation. DAPI (4′,6-diamidine-2-phenindoles) staining was used to identify the cell bodies. Finally, 10% of glycerine was used to seal the slides [15]. To evaluate morphology, sections were Nissl-stained with Cresylviolet (C0117, Beyotime).

Fluorescent images were acquired with an Olympus vs 120 microscope and a Nikon CSU Sora 2Camera confocal microscope. Brightfield images were taken using an Olympus vs 120 microscope. All images were analyzed with ImageJ and Qupath software. Methods of cell quantitative analysis have been described in the Additional file 1.

#### Western blot analysis

Hippocampus tissues of mice were used for western blotting using previously described methods [16]. Equal amounts of protein were separated by 10% SDS–polyacrylamide gel electrophoresis and transferred onto nitrocellulose membranes. Membranes were blocked via 5% skim milk powder in Tris-buffered saline including 0.05% (v/v) Tween 20 (TBST) for 2 h at 25 °C and then incubated overnight with the primary antibodies to NMDAR1(1:1000, #32-0500, Invitrogen), synaptophysin (1:1000, ab32127, abcam), PSD-95 (1:1000, ab238135, abcam) and β-actin (1:1000, TA-09, ZSGB-BIO). Membranes were washed thrice with TBST over 15 min and incubated with secondary antibodies (ZSGB-BIO, Beijing, China) in 5% skim milk powder in TBST. The membranes were exposed to BCIP/NBT alkaline phosphatase color developing reagent (Beyotime Institute of Biotechnology, Shanghai, China) for 15 min. Bands corresponding to the proteins of interest were scanned and band density analyzed using the Quantity One automatic imaging analysis system (Bio-Rad).

To examine the synaptic effects of anti-IgLON5 antibodies, rat hippocampal neuron cultures were exposed to anti-IgLON5 IgG and healthy control IgG for 7 days. Primary cell cultures of rat hippocampal neurons were prepared from embryonic P18 day as previously reported [6]. Western blot analysis was carried out on hippocampal neuron cultures using previously described methods [17]. Total protein was extracted from cells using Cell Total Protein Extraction Kit (Sangon Biotech). The methods have been described in detail in the Additional file 1.

#### In vitro electrophysiological recordings

Whole-cell recordings were obtained from CA1 pyramidal neurons in acute brain slices from mice that had been stereotaxically injected with patient IgG or control IgG for seven days. Procedures for electrophysiological recordings followed the previous publications [15]. The electrophysiological recordings are detailed in the Additional file 1.

#### Recording and analysis of local field potentials

Local field potentials (LFP) recordings were performed following previous studies, with only minor modifications [17]. The mice were deeply anesthetized with isoflurane, and a 75-μm stainless-steel electrode (#791000, A-M system, USA) was subsequently placed in the principal cell layer of dorsal CA1 of the HPC. After surgery, the mice had at least 3 days to recover. Recording signals (low-pass filter: 1–100 Hz) were digitized by the Ephyslab System (Thinker Tech Nanjing Biotech Co. Ltd.) at 30 kHz and then resampled at 1 kHz for the LFP analysis. The videos were recorded simultaneously with a camera. Using MATLAB R2018b program for LFP data analysis, the recorded LFPs were filtered by a 50-Hz notching filter to remove the powerline artifact. The time window of the NOR event was defined as the period 1 s before and 2 s after the nose-poke of the new object. The normalized power change of the theta (4–12 Hz), beta (12–25 Hz), and gamma (25–90 Hz) rhythm was defined as the average power within 2 s after exploration divided by the average power within 1 s before exploration (1 s before the time window of the NOR event).

#### RNA extraction and RNA sequencing

Hippocampal tissue was dissected after euthanasia. The hippocampus tissue was collected into the RNase-free tube and kept frozen at − 80 °C until analysis. Total RNA was isolated and purified using TRIzol reagent (Invitrogen, Carlsbad, CA, USA). RNA amount and purity of each sample were assessed using NanoDrop ND-1000 (NanoDrop, Wilmington, DE, USA). RNA integrity was assessed by Bioanalyzer 2100 (Agilent, CA, USA) with RIN number > 7.0 and confirmed by electrophoresis with denaturing agarose gel. The transcriptome-seq was performed in LC-Bio, Hangzhou, China. About 10 µg total RNA was purified and poly(A) RNA was isolated. After purification, the Poly (A) RNA was reverse transcribed into small fragments to form cDNA library. Then the DNA was screened and purified by UDG enzyme digestion and PCR to obtain the final sequencing library. Illumina Hiseq4000 was used to sequence the library after qualified quality inspection according to the recommended protocols [18].

The differentially expressed mRNAs were selected with fold changes ≥ 2 or fold changes ≤ 0.5 and P-value < 0.05 by R package edgeR or DESeq2, and then the differentially expressed mRNAs were enriched by Gene Ontology (GO) and Kyoto Encyclopedia of Genes and Genomes (KEGG).

### Statistical analyses

All statistical analyses were performed in GraphPad Prism 8 or MATLAB R2018b programs. Statistical differences between groups were assessed using unpaired two-tailed Student’s *T*-test and two-way ANOVA followed by Sidak’s multiple comparison test. Differences between groups were judged to be statistically significant when *p* < 0.05. Definition of statistical significance: *p < 0.05; **p < 0.01; ***p < 0.001; ****p < 0.0001.

## Results

### Patient data

In this study, we used purified IgG from serum samples of one patient with anti-IgLON5 disease and one healthy person without detectable antineuronal autoantibodies (control IgG). A consistent finding across all antibody-mediated CNS diseases is that antibody levels in the serum are much higher than in the CSF [19]. Thus, we selected serum antibodies for animal experiments [11]. We previously found that cognitive impairment was a predominant clinical feature [20]. As such, we explored the mechanism of its occurrence.

**Fig. 1:**
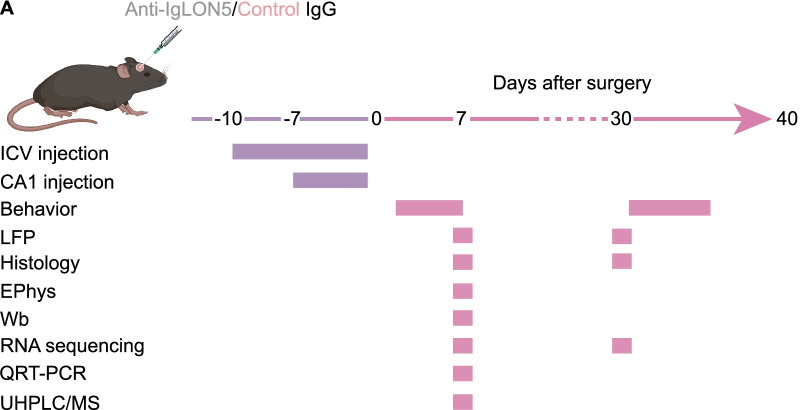
Experimental design. **A** Experimental design at different time points. *ICV* intra-cerebroventricular injection, *LFP* local field potentials, *Wb* western blot, *EPhys* electrophysiology, *QRT-PCR* quantitative real-time PCR, *UHPLC/MS* ultrahigh performance liquid chromatography–mass spectrometry

The main manifestations of the patient were cognitive impairment and anxiety, accompanied by mild sleep disorders. She had very high titers of IgLON5 IgG in the serum and cerebrospinal fluid via immunofluorescence assay (Additional file 2: Fig. S1A). She carried the HLA DRB1*10:01-DQB1*05:01 haplotype, which is present in approximately 60–70% of patients with IgLON5 antibodies [21]. Cranial ^18^F-fluorodeoxyglucose positron emission tomography magnetic resonance imaging (^18^F-FDG PET-MR) indicated mild hypometabolism in the left temporal lobe (Additional file 2: Fig. S1B). The timeline showing disease symptom progression, paraclinical examinations, and treatments over a 2-year follow-up is shown in Additional file 2: Fig. S1D. The experimental design is illustrated in Fig. 1A.

### Patient anti-IgLON5 antibodies cause cognitive impairments and behavior alternations in mice

**Fig. 2:**
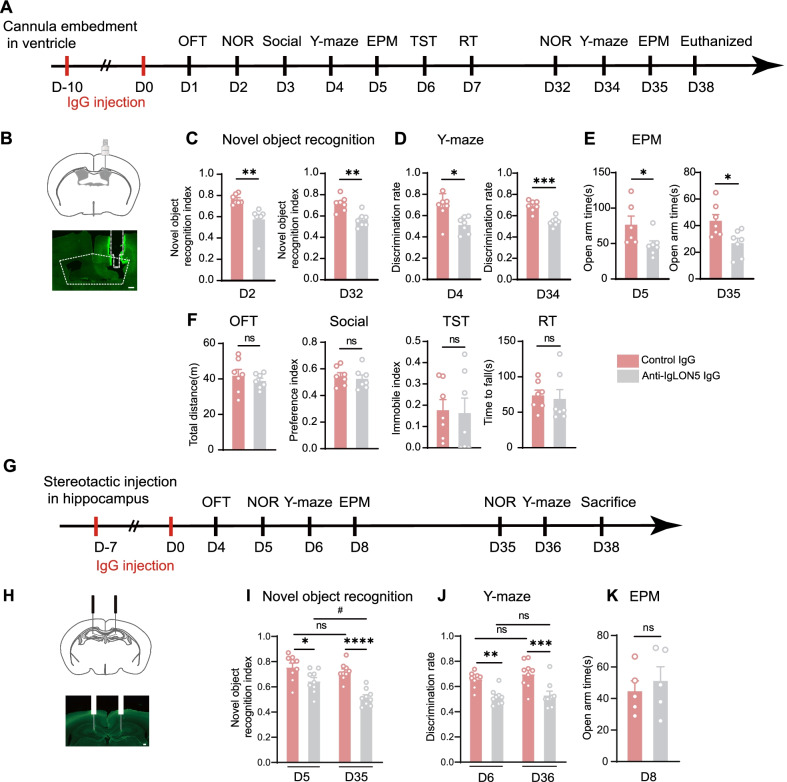
Infusion of antibodies in different passive-transfer models causes cognitive deficits in mice. **A** A unilateral cannula is implanted, and patient or control IgG is infused continuously in the ventricle from day -10 to day 0. Behavioral tasks are performed. **B** Schematic diagram of intraventricular cannula implantation. Scale bar = 250 µm. **C** The novel object recognition index is lower in IgLON5-IgG-injected mice than in healthy control IgG-injected mice (*t* = 3.776, *df* = 12, *p* = 0.0026, unpaired *t*-test), and there are differences in the novel object recognition index after 30 days (*t* = 4.214, *df* = 12, *p* = 0.0012, unpaired *t*-test). **D** In the Y-maze, there is a reduced discrimination rate (*t* = 3.007, *df* = 12, *p* = 0.0109, unpaired *t*-test) between the two groups, and the differences persist after 30 days (*t* = 5.715, *df* = 12, *p* < 0.0001, unpaired *t*-test). **E** In the EPM test, there is a decrease in open arm time (*t* = 2.234, *df* = 11, *p* < 0.05, unpaired *t*-test), and the differences persist after 30 days (*t* = 2.833, *df* = 12, *p* = 0.0151, unpaired *t*-test). **F** There are no differences in total distance, preference index, immobile index, or time to fall. **G** Experimental time course of the intrahippocampal injection passive-transfer model. Stereotactic microinjections of patient or control IgG into the dorsal CA1 of the hippocampus from day -7 to day 0. Behavioral testing is performed at the indicated time points. **H** Schematic diagram of stereotactic injection in hippocampus. Scale bar = 200 µm. **I** The novel object recognition index is lower in IgLON5-IgG-injected mice than in healthy control IgG-injected mice (*p* = 0.0145, two-way ANOVA), and there are differences in the novel object recognition index after 30 days (*p* < 0.0001, two-way ANOVA). **J** In the Y-maze, there is a reduced discrimination rate (*p* = 0.0023, two-way ANOVA), and the differences persist after 30 days (*p* = 0.0004, two-way ANOVA). **K** There are no significant differences in EPM test. Statistical significance was determined using the unpaired two-tailed Student’s *t*-test. **I** and **J** used the repeated measure two-way ANOVA followed by Sidak’s multiple comparison test. For **B**–**F**, *n* = 7 per group. For **G**–**K**, *n* = 9 per group. For (**K**), *n* = 5 per group. *^,#^*p* < 0.05, **p < 0.01, ***p < 0.001, ****p < 0.0001versus control IgG group. D = day

To investigate the functional effects of anti-IgLON5 antibodies on animal behavior, we used two independent passive-transfer animal models: (1) continuous 10-day infusion of patient IgG or control IgG directly into the lateral ventricle [9, 22, 23]; (2) stereotactic injections of patient IgG or control IgG directly into the CA1 (cornu ammonis 1) region of the hippocampus on both sides [24, 25] (Fig. 2A, G).

In the first model of 10-day intraventricular IgG infusion, behavioral tests were conducted for 7 days following the final injection (days 1–7) and for another 3 days on days 32–35. The most robust effects on Days 1–7 were presented in the novel object recognition test and *Y*-maze test (NOR: *t* = 3.776, *df* = 12, *p* = 0.0026; *Y*-maze: *t* = 3.007, *df* = 12, *p* = 0.0109) (Fig. 2C, D). The anti-IgLON5-IgG infused mice showed a progressive decrease in the object recognition index and discrimination rate (NOR: *t* = 4.214, *df* = 12, *p* = 0.0012; *Y*-maze: *t* = 5.715, *df* = 12, *p* < 0.0001), indicating long-term and short-term working memory deficit.

In addition, the elevated plus maze test results showed that intraventricular injection of patient IgG caused the mice to develop anxiety-like behavior (*t* = 2.234, *df* = 11, *p* < 0.05), which was manifested by a shorter period in the open arm, and this anxiety-like behavior persisted after 30 days (*t* = 2.833, *df* = 12, *p* = 0.0151) (Fig. 2E). Importantly, memory deficit and anxiety in anti-IgLON5 IgG infused mice persisted until day 32–35, indicating the long-term effects of IgLON5 antibodies. Results of locomotor activity (open field test and rotarod test), depression (tail suspension test), and social activity (three-chamber social interaction test) tests showed no significant differences between the two groups (Fig. 2F).

Considering that cognitive impairment was the most significant behavioral change observed after the intraventricular injection of antibodies, we stereotactically injected antibodies locally in the CA1 region of the hippocampus, the brain region most closely associated with cognition, and observed the behavioral changes. Consistently, the anti-IgLON5 IgG-injected mice showed impaired cognition in the novel object recognition test and Y-maze test on day 5 and 7 (NOR: *F* (1, 16) = 6.791, *P* = 0.0145; *Y*-maze: *F* (1, 16) = 0.7811, *P* = 0.0023). Notably, we found a significant deterioration of memory function in anti-IgLON5 IgG-injected mice with longer recovery around 30 days later (day 35 and 36) (NOR: *F* (1, 16) = 46.95, *P* < 0.0001; *Y*-maze: *F* (1, 16) = 39.46, *P* = 0.0004) (Fig. 2I, J). The results of anxiety-like behaviors (elevated plus maze) showed no differences between the two groups (Fig. 2K).

To obtain a comprehensive measurement, we also examined the effect of anti-IgLON5 IgG on metabolism in mice. On recording the body weight, water intake, food intake, and anal temperature of mice, we found that some indices changed after the final injection (Additional file 3: Fig. S2C–F).

These results indicate that our two independent passive-transfer animal models for in vivo application of anti-IgLON5 autoantibodies induced typical signs of anti-IgLON5 disease in the recipient mice.

### Dysfunction of memory-related neuronal oscillations in the CA1 of patient IgG-injected mice

**Fig. 3:**
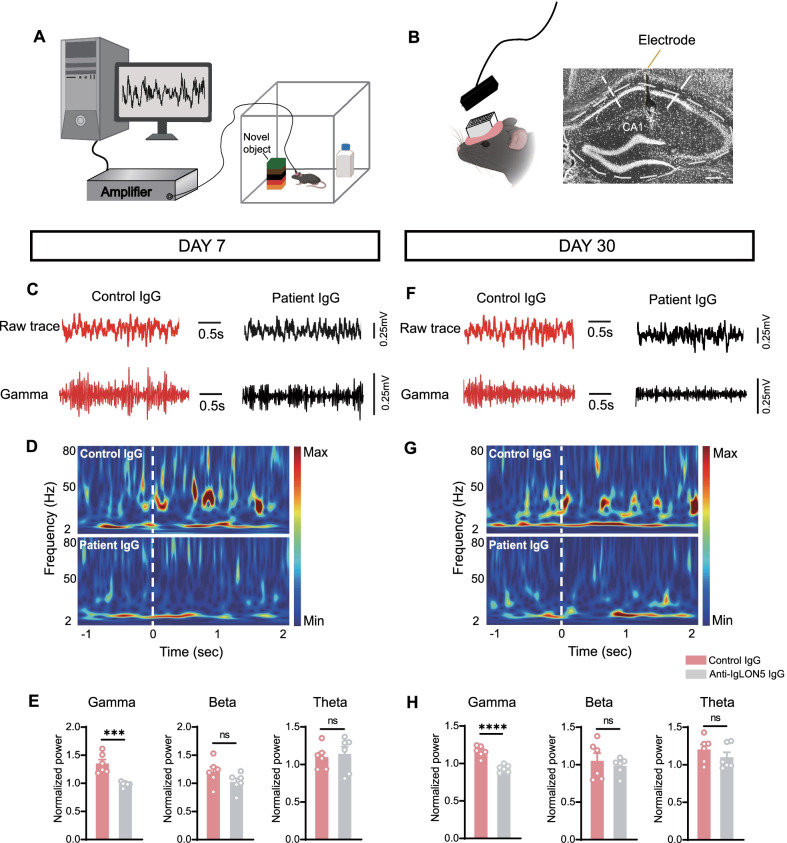
Dysfunction of memory-related neuronal oscillations in the CA1 of patient IgG-injected mice. **A**, **B** Schematic of the experimental design. Scale bar = 200 μm. **C** Time course of CA1 raw LFP from a mouse in the control or patient group exploring freely at day 7. **D** During novel object recognition task at day 7. The time “0” refers to the onset of an interaction event. Warmer colors indicate enhanced power. **E** Mean theta, beta, and gamma power while the mice explore the novel object at day 7. The patient IgG-injected mice show significantly lower gamma-band activity than does the healthy control IgG-injected mice (*t* = 4.941, *df* = 10, *p* = 0.0006; unpaired *t*-test). **F** Time course of CA1 raw LFP from a mouse in the control or patient group exploring freely at day 30. **G** During novel object recognition task at day 30. The time “0” refers to the onset of an interaction event. Warmer colors indicate enhanced power. **H** Mean theta, beta, and gamma power while the mice explore the novel object at day 30. The patient IgG-injected mice show significantly lower gamma-band activity than does the healthy control IgG-injected mice at day 30 (*t* = 6.269, *df* = 10, *p* < 0.0001, unpaired *t*-test). For **C**–**H**, *n* = 6 per group. ***p < 0.001, ****p < 0.0001 versus control IgG group

Oscillatory fluctuations of local field potentials (LFPs) in the theta (4–8 Hz) and gamma (25–90 Hz) bands play a mechanistic role in various aspects of memory including the encoding, consolidation, and retrieval of episodic memories [26]. As abnormalities in the novel object recognition test occurred in patient IgG-injected mice, we recorded the LFPs from the CA1 of the dorsal hippocampus when the mice performed the NOR test using in vivo electrophysiological recordings (Fig. 3A, B). The power ratio in the 1 s before and 2 s after exploring the novel object were measured in both the anti-IgLON5 IgG-injected mice and the control group. The normalized power values of the gamma, beta, and theta bands were calculated. At day 7, during novel object recognition (touching or sniffing the object), the patient IgG-injected mice showed significantly lower gamma band activity than did the control IgG- injected mice (*t* = 4.941, *df* = 10, *p* = 0.0006) (Fig. 3C–E). At 30 days after discontinuation of IgG, the differences in gamma-band activity between patient IgG- injected mice and the control group persisted (*t* = 6.269, *df* = 10, *p* < 0.0001) (Fig. 3F–H). These results indicate that anti-IgLON5 IgG causes gamma-oscillation dysfunction in mice.

### Anti-IgLON5 antibodies bind the brain for a long time and caused neuronal loss in vivo

**Fig. 4:**
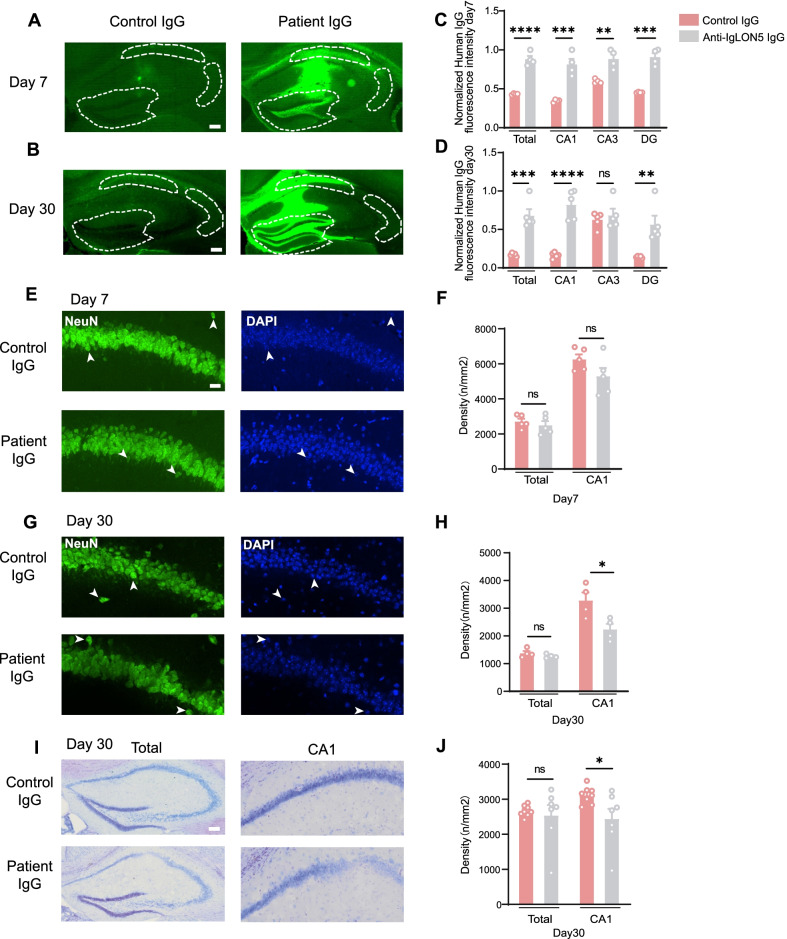
Anti-IgLON5 IgG bind in vivo for a long time and cause damage to neurons in the hippocampus. **A** Representative images of IgG binding to the hippocampus in perfused fixed brains on day 7. Scale bar = 100 µm. **B** Representative images of IgG binding to the hippocampus in perfused fixed brains on day 30. Scale bars = 100 µm. **C** Quantification of the intensity of human IgG immunolabeling in the hippocampus of mice injected with patient IgG or control IgG killed on day 7 (total: *t* = 10.27, *df* = 6, *p* < 0.0001; unpaired *t*-test; CA1: *t* = 0.0007, *df* = 6, *p* = 0.0007; unpaired *t*-test; CA3: *t* = 4.691, *df* = 6, *p* = 0.0034; unpaired *t*-test; DG: *t* = 8.742, *df* = 6, *p* = 0.0001; unpaired *t*-test). **D** Quantification of the intensity of human IgG immunolabeling in the hippocampus of mice infused with patient IgG or control IgG killed on day 30 (total: *t* = 6.280, *df* = 8, *p* = 0.0002; unpaired *t*-test; CA1: *t* = 7.678, *df* = 8, *p* < 0.0001; unpaired *t*-test; CA3: *t* = 0.6918, *df* = 8, *p* = 0.5086; unpaired *t*-test; DG: *t* = 3.885, *df* = 8, *p* = 0.0046; unpaired *t*-test). **E** Representative pictures of NeuN expression in the hippocampus of mice injected with patient IgG or control IgG killed on day 7. Scale bars = 20 μm. The white arrows on the left and right represent the same neuron. **F** Quantification of NeuN-positive cell density in the total hippocampus and CA1 region on day 7. **G** Representative pictures of NeuN expression in the hippocampus of mice on day 30. Scale bars = 20 μm. The white arrows on the left and right represent the same neuron. **H** Quantification of NeuN-positive cell density in the total hippocampus and CA1 region on day 30 (*t* = 2.935, *df* = 6, *p* = 0.0261; unpaired *t*-test). **I** Representative pictures of Nissl-stained sections in the hippocampus of mice injected with patient IgG and control IgG and killed on day 30. Scale bars = 100 μm. **J** Quantification of Nissl-stained positive cell densities in the total hippocampus and CA1 regions on day 30 (CA1: *t* = 2.705, *df* = 13, *p* = 0.018; unpaired *t*-test; Total: *t* = 0.4548, *df* = 13, *p* = 0.6568; unpaired *t*-test). For **A**–**D**, *n* = 4 or 5 per group. For (**E**)–(**H**), *n* = 4 or 5 per group. For **I**–**J**, *n* = 7 per group. *p < 0.05, **p < 0.01, ***p < 0.001, ****p < 0.0001 vs control IgG group. NeuN = neuronal nuclear antigen

Neuropathological autopsy of some anti-IgLON5 cases showed gliosis and neuronal loss in the brain stem, tegmental, hypothalamus and hippocampus [27]. To further demonstrate the pathogenicity of antibodies against anti-IgLON5 disease, brain tissue was analyzed for human IgG and neuronal markers. Mice injected with anti-IgLON5 IgG, but not control IgG, had progressively increased human IgG immunostaining (representing IgG bound to brain) at day 7, especially in the CA1 and DG regions (Total: *t* = 10.27, *df* = 6, *p* < 0.0001; CA1: *t* = 0.0007, *df* = 6, *p* = 0.0007; CA3: *t* = 4.691, *df* = 6, *p* = 0.0034; DG: *t* = 8.742, *df* = 6, *p* = 0.0001) (Fig. 4A, C). In addition, the amount of human IgG bound to all selected regions of the hippocampus was significantly higher in the anti-IgLON5 IgG-injected mice than in the control group at 30 days after IgG injection was stopped (Total: *t* = 6.280, *df* = 8, *p* = 0.0002; CA1: *t* = 7.678, *df* = 8, *p* < 0.0001; CA3: *t* = 0.6918, *df* = 8, *p* = 0.5086; DG: *t* = 3.885, *df* = 8, *p* = 0.0046), suggesting that anti-IgLON5 antibody produced relatively long-term damage (Fig. 4B, D).

The densities of NeuN-positive neurons were measured in the hippocampus. No evidence of neuronal loss was found in the anti-IgLON5 IgG at day 7 (Fig. 4E, F). However, neuron density in the CA1 region was decreased 30 days after anti-IgLON5 antibody injection (*t* = 2.935, *df* = 6, *p* = 0.0261), while there was no difference in total neuron density in the hippocampus (Fig. 4G, H).

Both groups showed no gross morphological changes in Nissl-stained sections in the hippocampus after 7 days (data not shown). Nissl staining after 30 days of IgG injection showed that compared with the control group, there were some morphological changes in the neurons in CA1 region of the anti-IgLON5 group but not in the control group (CA1: *t* = 2.705, *df* = 13, *p* = 0.018; Total: *t* = 0.4548, *df* = 13, *p* = 0.6568), which was consistent with the results of NeuN staining of neurons (Fig. 4I, J).

### Anti-IgLON5 IgG-injected mice display microglial and astrocytic activation

**Fig. 5:**
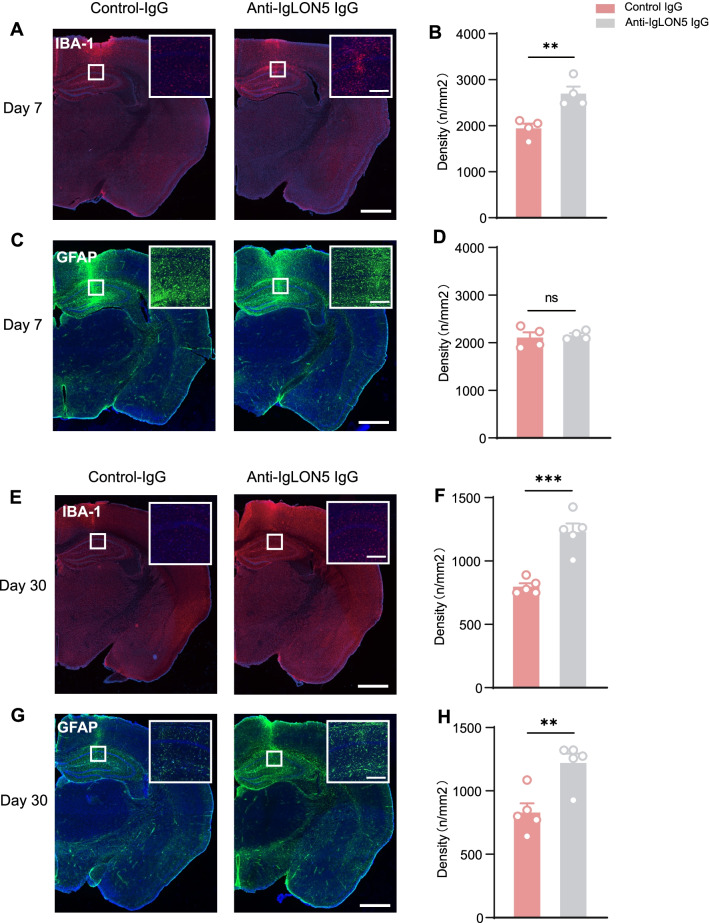
Anti-IgLON5 IgG-injected mice display microglial and astrocytic activation. **A** Immunofluorescence for IBA-1 (red) in anti-IgLON5 IgG-injected mice (right) compared to controls (left) on day 7. The higher magnification insets from the hippocampus show individual microglia. **B** Quantification of IBA-1 positive cell densities in the total hippocampus on day 7 (*t* = 4.169, *df* = 6, *p* = 0.0059; unpaired *t*-test). **C** Immunofluorescence for GFAP (green) in anti-IgLON5 IgG-injected mice (right) compared to controls (left) on day 7. The higher magnification insets from the hippocampus show individual astrocyte. **D** Quantification of GFAP-positive cell densities in the total hippocampus on day 7 (*t* = 0.3880, *df* = 6, *p* = 0.7114; unpaired *t*-test). **E** Immunofluorescence for IBA-1 (red) in anti-IgLON5 IgG-injected mice (right) compared to controls (left) on day 30. **F** Quantification of IBA-1 positive cell densities in the total hippocampus on day 30 (*t* = 5.966, *df* = 8, *p* = 0.0003; unpaired *t*-test). **G** Immunofluorescence for GFAP (green) in anti-IgLON5 IgG-injected mice (right) compared to controls (left) on day 30. **H** Quantification of GFAP-positive cell densities in the total hippocampus on day 30 (*t* = 3.752, *df* = 8, *p* = 0.0056; unpaired *t*-test). Scale bars = 1000 µm (Inset, 200 µm). For **A**–**D**, *n* = 4 per group. For **E**–**H**, *n* = 5 per group. **p < 0.01, ***p < 0.001 vs control IgG group

Activated microglial cells, as identified by expression of IBA1 (Fig. 5A), were measured in the hippocampus. Microglial density was increased in the hippocampus of anti-IgLON5 IgG-injected mice at Day 7 (*t* = 4.169, *df* = 6, *p* = 0.0059) (Fig. 5B). At Day 30, significant activation of microglia compared to the control-IgG group (*t* = 5.966, *df* = 8, *p* = 0.0003) (Fig. 5E, F) was seen. There were no evident differences in the density of GFAP-positive cells in the hippocampus at Day 7 (Fig. 5C, D), but increased GFAP expression in the hippocampus of anti-IgLON5 IgG-injected mice at Day 30 (*t* = 3.752, *df* = 8, *p* = 0.0056) (Fig. 5G, H). These microglial and astrocyte changes were suggestive of a state of neuroinflammation in the anti-IgLON5 IgG-injected mice.

### Anti-IgLON5 IgG exposure disrupted synaptic homeostasis

**Fig. 6:**
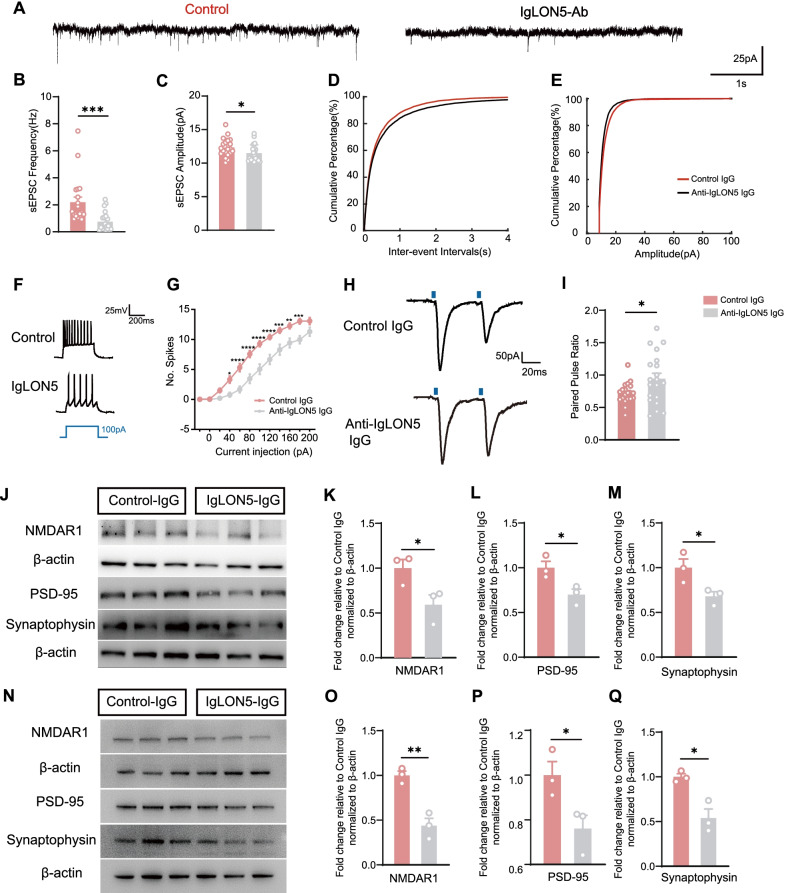
Anti-IgLON5 IgG exposure causes imbalance of synaptic homeostasis. **A** Example traces showing sEPSCs recorded in pyramidal neurons of CA1 for control IgG-injected and IgLON5 IgG-injected mice. **B** Average sEPSC frequency in the two groups (*t* = 3.578, *df* = 39, *p* = 0.0009, unpaired *t*-test). **C** Average sEPSC amplitudes in the two groups (*t* = 2.052, *df* = 39, *p* = 0.0469, unpaired *t*-test). **D** Cumulative probability of inter-event internal sEPSCs. Control: *n* = 20 neurons from four mice; IgLON5-Ab: *n* = 21 neurons from four mice. **E** Cumulative probability of the amplitude of sEPSCs. Control: *n* = 20 neurons from four mice; IgLON5-Ab: *n* = 21 neurons from four mice. **F** Current-clamp recordings of anti-IgLON5 IgG and Control-IgG group. **G** The number of spikes in anti-IgLON5 IgG and Control-IgG group (*n* = 24–28 neurons, *p* < 0.0001, two-way ANOVA). **H** Representative traces recorded in CA1 pyramidal cells from anti-IgLON5 IgG and Control-IgG hippocampal slices following paired-pulse stimulation protocol. **I** Averaged PPR from anti-IgLON5 IgG and Control-IgG group (*n* = 19–20 neurons, *t* = 2.265, *df* = 37, *p* = 0.0294, unpaired *t*-test). **J** Immunoblot analysis of PSD-95, synaptophysin, and NMDAR1 expression in the hippocampus of IgLON5-IgG- and HC-IgG-treated mice. **K–M** Quantification of PSD-95, synaptophysin, and NMDAR1 protein levels shows that the levels of these proteins are significantly lower in the IgLON5-IgG-injected group than in the control group. (NMDAR1: *t* = 2.785, *df* = 4, *p* = 0.0496, unpaired *t*-test; PSD-95: *t* = 3.163, *df* = 4, P = 0.0341, unpaired *t*-test; synaptophysin: *t* = 2.899, *df* = 4, *p* = 0.0441, unpaired *t*-test). **N** Immunoblot analysis of PSD-95, synaptophysin, and NMDAR1 expression in cultures exposed to anti-IgLON5 IgG. **O–Q** Quantification of PSD-95, synaptophysin, and NMDAR1 protein levels showed that the expressions of these proteins are significantly lower in cultures exposed to anti-IgLON5 IgG than in the control group (NMDAR1:*t* = 5.835, *df* = 4, *p* = 0.0043, unpaired *t*-test; PSD-95: *t* = 2.828, *df* = 4, P = 0.0474, unpaired *t*-test; synaptophysin: *t* = 4.310, *df* = 4, *p* = 0.0126, unpaired *t*-test). For **B**–**E**, *n* = 20 or 21. For **F**–**G**, *n* = 24–28 per group. For **H**–**I**, *n* = 19 or 20 per group. For **J**–**Q**, *n* = 3 per group. *p < 0.05, **p < 0.01, ***p < 0.001, ****p < 0.0001 vs control IgG group

To investigate whether anti-IgLON5 IgG injection affects synaptic transmission, we recorded the spontaneous excitatory post-synaptic currents (sEPSCs) of pyramidal neurons in the CA1 region by whole-cell patch-clamp recording of acute brain slices of IgLON5-IgG-injected mice and control mice at day 7 (Fig. 6A). We found that the frequency and the amplitude of sEPSCs decreased significantly in anti-IgLON5 IgG-injected mice (frequency: *t* = 3.578, *df* = 39, *p* = 0.0009; amplitude: *t* = 2.052, *df* = 39, *p* = 0.0469) (Fig. 6B, C). The action potential was measured and showed a decrease in the evoked spikes after anti-IgLON5 IgG injection compared to those in the control group (Fig. 6F, G). Paired-pulse facilitation is a form of short-term synaptic plasticity, which reflects the release probability of the presynaptic cell. Greater facilitation in the paired pulse ratio (PPR) indicates decreased release probability. In this condition, a higher PPR was noted in anti-IgLON5 IgG-injected mice (*t* = 2.265, *df* = 37, *p* = 0.0294) (Fig. 6H, I), suggesting a presynaptic defect associated with lower release probability. The UHPLC–MS method was employed to quantify the three main neurotransmitters in the hippocampus: Glu, GABA, and NE. The glutamate level in the hippocampus of the anti-IgLON5 IgG-injected group was lower than that of the control group. However, there were no significant differences in the other two neurotransmitters (Additional file 5: Fig. S4A–C).

To further examine the synaptic mechanisms of antibody injection, IHC analysis for the expression of presynaptic markers, synaptophysin and post-synaptic markers, post-synaptic density protein (PSD)-95, and NMDAR1 were performed in the hippocampal CA1 region (Fig. 6J). Western blot analysis showed a marked decrease in PSD-95, synaptophysin, and NMDAR1 protein levels in the hippocampus of IgLON5-IgG-injected mice compared with those in control mice at day 7 (NMDAR1:*t* = 2.785, *df* = 4, *p* = 0.0496; PSD-95: *t* = 3.163, *df* = 4, P = 0.0341; synaptophysin: *t* = 2.899, *df* = 4, *p* = 0.0441; the full blot is provided in Additional file 6: Fig. S5A) (Fig. 6K–M). In parallel experiments, we quantified the amount of post-synaptic and presynaptic proteins NMDAR1, PSD95, and synaptophysin (Fig. 6N). All were found to be decreased in cultures exposed to anti-IgLON5 IgG (NMDAR1:*t* = 5.835, *df* = 4, *p* = 0.0043; PSD-95: *t* = 2.828, *df* = 4, *p* = 0.0474; synaptophysin: *t* = 4.310, *df* = 4, *p* = 0.0126; the full blot is provided in Additional file 6: Fig. S5B) (Fig. 6O–Q).

Electrophysiological measurements and western blot results collectively showed that anti-IgLON5 IgG injection resulted in synaptic homeostasis imbalance through decreased excitation, affecting synaptic function.

### Whole-genome transcriptomic analyses

**Fig. 7:**
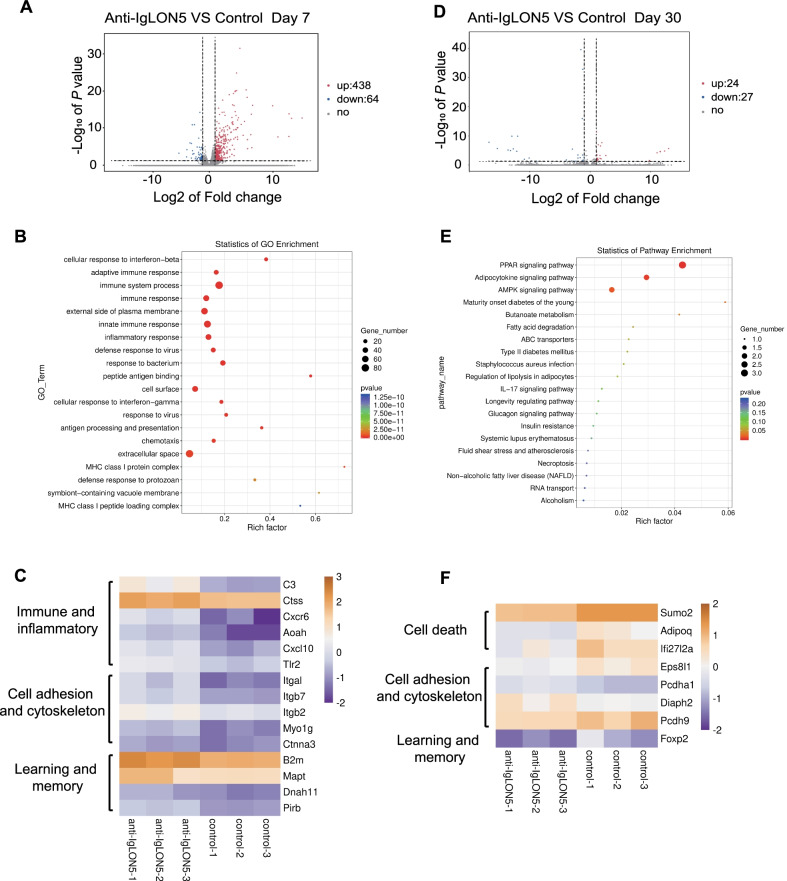
Whole-genome transcriptomic analyses. **A** Volcano plot of differentially expressed genes between IgLON5-IgG-injected mice and HC-IgG-injected mice at day 7. Downregulation and upregulation are shown in blue and red dots, respectively. **B** Statistics of GO enrichment on day 7. Rich factor = S gene number/B gene number. The size of dots represents the S gene number, and the color of dots represents the *p* value. S gene, significant differentially expressed gene annotated as specific GO term; B gene, gene annotated as a specific GO term. **C** Cluster analysis of high expression levels of differentially expressed genes on day 7. There are three main categories: immune and inflammatory, cell adhesion and cytoskeleton, and learning and memory. **D** Volcano plot of differentially expressed genes between IgLON5-IgG-injected mice and HC-IgG-injected mice at day 30. **E** KEGG analyses of RNA-seq data at day 30 showing the top 20 regulated pathways in IgLON5-IgG-injected mice in comparison with control-IgG-injected mice. The P-value represents the level of significance of enrichment. **F** Cluster analysis of high expression levels of differentially expressed genes on day 30. There are three main categories: cell death, cell adhesion and cytoskeleton, and learning and memory. For **A**–**F**, *n* = 3 per group

We performed RNA-seq analyses of patient IgG- and control IgG-treated mice at day 7 and day 30 after discontinuation of IgG to explore the potential molecular mechanisms. RNA was extracted from the hippocampal tissues. At day 7, there were 502 differentially expressed genes (DEGs) between anti-IgLON5 IgG and control IgG-injected mice, of which 438 genes were upregulated and 64 genes were downregulated (*p* value < 0.05 and absolute log2 (fold change) > 1). To show the general scattering of the genes and to filter the DEGs, volcano plots were constructed (Fig. 7A). The distribution of significant DEGs was reflected through the Gene Ontology (GO) enrichment scatter plot (three terms: biological process, cellular component, and molecular function) (Fig. 7B).

We then focused on the relatively high expression levels of DEGs for different functions (Fig. 7C). As expected, most of the gene function in inflammatory and immune responses, such as C3, Ctss, Cxcr6, Aoah, Cxcl 10, and Tlr2. Some differential genes also played a role in cell adhesion and cytoskeleton, as IgLON5 is well-known as a cell adhesion molecule. There are also several genes implicated in learning and memory that are relevant to our research. QRT-PCR was used to further verify some genes suggested by RNA-Seq (Additional file 6: Fig. S5C). Related inflammatory markers and chemokines were detected: TGF-β, IL-6, IL-1-β, TNF-α, CXCL16, CXCR6, CCL5, CCL12, CXCL13. The relative mRNA expression of TGF-β, CCL5, and CXCL13 was found to be increased in the anti-IgLON5 group compared to that of the control-IgG group 7 days after cessation of human IgG injection.

However, on day 30, we found 51 DEGs between anti-IgLON5 IgG- and control IgG-injected mice, of which 24 genes were upregulated and 27 genes were downregulated (Fig. 7D). In addition, a biological pathway distribution was observed in Kyoto Encyclopedia of Genes and Genomes enrichment analysis (Fig. 7E). There were various signaling pathways, including PPAR, adipocytokine, and AMPK. The results of the heat map suggested that DEGs at day 30 were associated with cell death, cell adhesion and cytoskeleton, learning, and memory (Fig. 7F).

## Discussion

To our best knowledge, this is the innovative study to show that anti-IgLON5 antibodies cause disease symptoms in vivo by directly injecting anti-IgLON5 antibodies into the mouse brain. The pathogenicity of anti-IgLON5 antibodies provides a unique and disease-related concept of direct immune–neuronal interaction to clarify the symptoms of this disease and impaired memory formation in patients. Further, our results identify a unique immune–neuronal interaction that may underlie the characteristic disease symptoms.

### Relatively long-term effects of anti-IgLON5 antibodies in vivo

Anti-IgLON5 disease usually has a protracted clinical course and is diagnosed many months after symptom onset in contrast to anti-NMDAR or anti-AMPAR encephalitis that usually has a rapid presentation in days or weeks [27, 28]. Considering that animal models of anti-NMDAR encephalitis can recover from related behavioral damage 14 days after discontinuation of antibody injection [29, 30], while this type of disease still has behavioral damage 30 days after discontinuation of antibody injection, the current study established the relatively long-term effects of anti-IgLON5 antibodies. Continuous application of anti-IgLON5 antibodies with cannula embedment and repeated microinjection both consistently afflicted memory and cognition for a relatively long time. In addition, mice injected with anti-IgLON5 IgG embedded in the ventricles showed long-term anxiety-like behavior, which was consistent with the patient’s clinical characteristics.

We observed deposition of human IgG in the hippocampus for a relatively long time and damage of neurons after 30 days of anti-IgLON5 antibody injection. Neuronal damage was progressive and no evident neuronal damage was observed at 7 days after discontinuation of antibody injection, consistent with what has been observed in other studies: There was no difference in cell death for human-induced pluripotent stem cell (hiPSC)-derived cultures after 7 days of exposure to anti-IgLON5 IgG, but significant cell death was observed after 21 and 35 days [31]. Progressive neuronal injury might be accompanied by an initial cytoskeletal disruption and neuroinflammatory reactions [7].

### Synaptic plasticity as a potential mechanism in anti-IgLON5 disease

Our results show that patient antibodies reduced the electrophysiological activity of neurons. Although this has never been previously shown as an effect of anti-IgLON5 antibodies, changes in electrophysiological properties are a commonly described effect of antibodies of other subtypes of autoimmune encephalitis [32–34]. In our model, the reduced frequencies and amplitudes of sEPSCs recorded by pyramidal neurons, decreases in the spike of the action potential, and increases in PPR suggest that the presynaptic release probability and the number of functional excitatory synapses are downregulated, which may lead to inappropriate excitatory/inhibitory balance in local HPC circuits. Therefore, these autoantibodies, contribute to cognitive deficits by directly targeting neuronal molecules that regulate synaptic excitability [35].

The results showed many clusters of post- and pre-synaptic proteins (PSD95, NMDAR1, and synaptophysin (SYN)), and these clusters were significantly reduced in rat hippocampal neurons exposed to anti-IgLON5 IgG. These findings are in agreement with a previous report showing that the amount of post- and pre-synaptic proteins PSD95 and synaptophysin were decreased in hiPSC derived cultures exposed to anti-IgLON5 IgG [31].

Cognitive impairment is associated with synaptic abnormalities in the hippocampus [36]. Pre-synaptic and post-synaptic proteins such as SYN and PSD-95, respectively, play important roles in synaptic plasticity and cognitive function [37]. SYN protein levels are lower in elderly individuals with dementia. Recent, evidence shows that PSD-95 disruption is associated with cognitive and learning deficits observed in schizophrenia and autism [38]. NMDA receptors also play a dominant role in synaptic plasticity and are responsible for working memory function and cognitive performance. This indicates that IgLON5 antibodies may lead to an imbalanced synaptic homeostasis and impaired synaptic function, which may provoke pathological excitatory neurotransmission. These deficits in synaptic plasticity most likely account for the behavioral abnormalities observed in passive-transfer models.

### Impairment of gamma-oscillations may lead to cognitive impairment

The rhythmic activity of neuronal populations oscillates in the brain’s LFP, which plays a functional role in neural synchronization mechanisms, abnormalities of which have been implicated in the pathophysiology of many neuropsychiatric disorders [39]. Changes in neural oscillations in various physiological rhythms are associated with cognitive performance. Theta and gamma rhythms are two prevalent rhythms in the hippocampus and are believed to be extremely relevant to cognition [40]. In our study, anti-IgLON5 IgG-injected mice displayed aberrant gamma power in the CA1 during novel object recognition testing at 7 and 30 days after the end of antibody injection. By using LFP recording, the gamma power provides information about the collective behavior of neurons and the mode of operation of a given network [41]. Our data extend previous findings that gamma-oscillation deficits play a vital role in autoimmune induced cognitive impairments [42].

Oscillatory activity plays an important role not only within local neuronal networks, but also in the integration of coordinated activity among distant brain regions [43]. The hippocampus, prefrontal cortex, and interconnected neural circuits are implicated in several aspects of cognitive and memory processes [44]. Therefore, we hypothesized that impaired memory consolidation mechanisms of the hippocampal–cortical neural circuit may be responsible for spatial memory deficits [45]. This mechanism could be further studied in the future [46].

### Anti-IgLON5 antibody may cause neuroinflammation

An increase in the expression of microgliosis and astrogliosis markers in the hippocampus was noted. Previous studies have shown that activated microglia can induce reactive astrocytes under pathological conditions, which upregulate C3 expression and are responsible for neurotoxicity and synaptic loss [47, 48]. Additionally, microglial activation has been reported in neuropathological cases of patients with anti-IgLON5 disease [49]. In a recent case series study, anti-IgLON5 patients showed inflammatory changes and increased B lymphocyte frequency on routine cerebrospinal fluid analysis, which supports the presence of an inflammatory response [50]. This study also found that the relative mRNA expression of TGF-β, CCL5, and CXCL13 increased in the anti-IgLON5 group compared to that of the control-IgG group 7 days after cessation of human IgG injection.

At the same time, evidence exists that supports the direct pathogenicity of anti-IgLON5 IgG. Firstly, neural adhesion proteins such as IgLON5 are crucial in the development and maintenance of functional neural connectivity. Altered expression of IgLON5 may cause suicidal behaviors in psychotic patients [51]. Patients' antibodies can cause a decrease of cell surface IgLON5 clusters, which strongly indicates the pathogenicity of anti-IgLON5 antibody [6]. Secondly, anti-IgLON5 IgG that we purified was of high titers. Moreover, the anti-IgLON5 IgG did bind in the mouse brain for a longer period of time, rather than for a while, which indicated that anti-IgLON5 IgG took part in the pathological process continuously. Thirdly, post-mortem findings in two patients showed neuronal loss and predominantly involving the hypothalamus and tegmentum of the brain stem without evidence of inflammation [1, 3]. Fourthly, anti-IgLON5 IgG can disrupt the cytoskeletal organization in cultured rat hippocampal neurons, resulting in dystrophic neurites and axonal swelling without involvement of inflammation.

Based on the findings of this research, negative effects on neural activity may result from a combination of anti-IgLON5 antibodies and neuroinflammation. Further research is required to evaluate the pathogenicity of anti-IgLON5 antibodies via their direct function and antibody-induced inflammatory microenvironments.

The limitation of this study was related to the type of disease and symptoms in the model. Anti-IgLON5 disease results in a broader spectrum of symptoms, aside from memory and cognitive deficits [52]. Further experimental studies on the effects of anti-IgLON5 antibodies on sleep and movement disorders are needed. Moreover, only one case and one control sample were used in our study. Anti-IgLON5 disease is, rare, with an estimated prevalence of 1/150,000, and only about 10 cases have been documented in China. Although our research indicates that anti-IgLON5 antibody induced a relatively long-term pathogenic effect, we have not conducted longer follow-ups (e.g., 2–6 months). Nevertheless, because little is known about the role of CSF and serum antibodies in certain situations, we used serum antibodies with higher titers rather than CSF antibodies as in other autoimmune animal models [9, 53]. Further studies on the link between antibody-mediated autoimmunity and neurodegeneration would be valuable for validating the specificity.

## Conclusions

The data in the current study indicate several novel findings. (1) Anti-IgLON5 antibodies may affect synaptic stability and function, leading to progressive cognitive impairment in mice. (2) In the long term, anti-IgLON5 antibodies can damage the neurons and activate astrocytes and microglial in vivo. (3) Gamma-oscillations play a key role in controlling cognitive activity in anti-IgLON5 disease. (4) Anti-IgLON5 antibodies can cause neuroinflammation. The negative effects on neural activity may result from a combination of anti-IgLON5 antibodies and neuroinflammation. These findings support the pathogenic role of anti-IgLON5 IgG that antibodies causing neuronal dysfunction and abnormal behaviors and lead the way for more detailed studies on how and where antibodies act to cause clinical features.

## Supplementary Information

**Additional file 1:** Supplemental materials for the methodology section.

**Additional file 2: Fig. S1**. Clinical information of an anti-IgLON5 disease patient. Related to Figure 1. **(A)** Representative images of cell-based assays showing anti-IgLON5 antibodies in the serum from the patient but not in the serum from a healthy control. Scale bar=20μm.** (B)** The cranial 18F-FDG PET-MR image of the patient upon first admission. The standardized uptake value ratios (SUVR) of the left thalamus, right thalamus, and left temporal lobe are 1.12, 0.97, and 0.91, respectively. The cranial 18F-FDG PET-MR of the patient upon the second admission. The SUVRs of the bilateral thalamus and left temporal lobe are lower than they are 18 months ago (left thalamus, 0.97; right thalamus, 0.82; left temporal lobe, 0.70). **(C) **Immunoprecipitation results show the presence of these antibodies in patient but their absence in control serum. **(D) **Timeline of symptoms.

**Additional file 3**: **Fig. S2. **Minor pathogenicity dose of anti-IgLON5 IgG and Metabolism in mice. Related to Figure 2.** (A) **We diluted the patient's IgG by 10-fold and 100-fold with PBS respectively (0.23mg/ml and 0.023mg/ml). The novel object recognition index of 1/10 anti-IgLON5 IgG injected mice is lower than healthy control IgG-injected mice at Day 7 (n=6 per group, p=0.0419, one-way ANOVA with Tukey’s post-hoc test). **(B)** In the Y-maze, there is a reduced discrimination rate in 1/10 anti-IgLON5 IgG injected mice than the other two groups at Day 7 (Control-IgG vs. 1/10 anti-IgLON5 IgG: n=6 per group, p=0.001, one-way ANOVA with Tukey’s post-hoc test; 1/10 anti-IgLON5 IgG vs. 1/100 anti-IgLON5 IgG: n=6 per group, p=0.0097, one-way ANOVA with Tukey’s post-hoc test). **(C)** Body weights were tested every 2 days after final injection at the same time period and recorded again 30 days after the injection (Day 8: p=0.0193, unpaired t-test; Day 15: p=0.0406, unpaired t-test). **(D)** Rectal body temperature was measured every 2 days after final injection and recorded again 30 days after the injection. **(E)** Food intake were measured once every 2 days after final injection at the same time and recorded again 30 days after the injection (Day 13: p=0.0059, unpaired t-test). **(F)** Water intake were measured once every 2 days after final injection at the same time and recorded again 30 days after the injection (Day 13: p=0.0208, unpaired t-test). For (C)–(F), n=6 per group. *p < 0.05, **p < 0.01, ***p < 0.001 vs control IgG group

**Additional file 4:** **Fig. S3. **The lower magnification views of Fig.4 E and G. Related to Figure 4. **(A)** The lower magnification views of Fig.4 E and G have provided. In the representative pictures of NeuN expression in the hippocampus on day 7 (left), the black rectangles that appear at the corners of these images are areas that are not scanned by the confocal microscope. Because it is outside the target area, the analysis result is not affected. The white box area is the area we present in the results. Scale bars = 100µm.

**Additional file 5: Fig. S4. **Anti-IgLON5 antibodies reduce associated neurotransmitters. Related to Figure 6.** (A) **Concentration of Glu in the hippocampus of control and anti-IgLON5 IgG group (n=3 per group, t=2.822, df=4, p=0.0477, unpaired t-test).** (B) **Concentration of GABA in the hippocampus of control and anti-IgLON5 IgG group.** (C) **Concentration of NE in the hippocampus of control and anti-IgLON5 IgG group.** (D) **Cultures treated with anti-IgLON5 IgG showed a decrease in PSD-95. Scale bar=25μm.

**Additional file 6**: **Fig. S5. **Anti-IgLON5 antibodies reduce cell surface synaptic proteins. Related to Figure 6.** (A) **Immunoblot analysis of NMDAR1, PSD-95, and Synaptophysin expression in brains of anti-IgLON5 IgG and control-IgG treated mice. **(B)** Immunoblot analysis of NMDAR1, PSD-95, and Synaptophysin expression in cultures exposed to anti-IgLON5 IgG and control-IgG. **(C)** QRT-PCR results of related inflammatory markers and chemokines: TGF-β, IL-6, IL-1β, TNF-α, CXCL16, CXCR6, CCL5, CCL12, CXCL13 (TGF-β: n=3 per group, t=5.739, df=4, p=0.0046, unpaired t-test; CCL5: n=3 per group, t=18.49, df=4, p<0.0001, unpaired t-test; CXCL13: n=3 per group, t=3.707, df=4, p=0.0207, unpaired t-test). *p < 0.05, **p < 0.01, ***p < 0.001, ****p < 0.0001 vs control IgG group.

## Data Availability

The key data are included in this article and its Additional files.

## Abbreviations

IgG: Immunoglobulin G
PSD95: Post-synaptic density protein 95
NMDAR 1: *N*-Methyl d-aspartate receptor 1

## References

[1] Sabater L, Gaig C, Gelpi E, Bataller L, Lewerenz J, Torres-Vega E, Contreras A, Giometto B, Compta Y, Embid C (2014). A novel non-rapid-eye movement and rapid-eye-movement parasomnia with sleep breathing disorder associated with antibodies to IgLON5: a case series, characterisation of the antigen, and post-mortem study. Lancet Neurol.

[2] Gaig C, Iranzo A, Cajochen C, Vilaseca I, Embid C, Dalmau J, Graus F, Santamaria J. Characterization of the sleep disorder of anti-IgLON5 disease. Sleep. 2019; 42. 10.1093/sleep/zsz133 31198936

[3] Gelpi E, Hoftberger R, Graus F, Ling H, Holton JL, Dawson T, Popovic M, Pretnar-Oblak J, Hogl B, Schmutzhard E (2016). Neuropathological criteria of anti-IgLON5-related tauopathy. Acta Neuropathol.

[4] Erro ME, Sabater L, Martinez L, Herrera M, Ostolaza A, GarciadeGurtubay I, Tunon T, Graus F, Gelpi E (2020). Anti-IGLON5 disease: a new case without neuropathologic evidence of brainstem tauopathy. Neurol Neuroimmunol Neuroinflamm.

[5] Gaig C, Ercilla G, Daura X, Ezquerra M, Fernandez-Santiago R, Palou E, Sabater L, Hoftberger R, Heidbreder A, Hogl B (2019). HLA and microtubule-associated protein tau H1 haplotype associations in anti-IgLON5 disease. Neurol Neuroimmunol Neuroinflamm.

[6] Sabater L, Planaguma J, Dalmau J, Graus F (2016). Cellular investigations with human antibodies associated with the anti-IgLON5 syndrome. J Neuroinflammation.

[7] Landa J, Gaig C, Plaguma J, Saiz A, Antonell A, Sanchez-Valle R, Dalmau J, Graus F, Sabater L (2020). Effects of IgLON5 antibodies on neuronal cytoskeleton: a link between autoimmunity and neurodegeneration. Ann Neurol.

[8] Pruss H (2021). Autoantibodies in neurological disease. Nat Rev Immunol.

[9] Planaguma J, Leypoldt F, Mannara F, Gutierrez-Cuesta J, Martin-Garcia E, Aguilar E, Titulaer MJ, Petit-Pedrol M, Jain A, Balice-Gordon R (2015). Human *N*-methyl d-aspartate receptor antibodies alter memory and behaviour in mice. Brain.

[10] Wright S, Hashemi K, Stasiak L, Bartram J, Lang B, Vincent A, Upton AL (2015). Epileptogenic effects of NMDAR antibodies in a passive transfer mouse model. Brain.

[11] Giannoccaro MP, Menassa DA, Jacobson L, Coutinho E, Prota G, Lang B, Leite MI, Cerundolo V, Liguori R, Vincent A (2019). Behaviour and neuropathology in mice injected with human contactin-associated protein 2 antibodies. Brain.

[12] Li J, Lu C, Gao Z, Feng Y, Luo H, Lu T, Sun X, Hu J, Luo Y (2020). SNRIs achieve faster antidepressant effects than SSRIs by elevating the concentrations of dopamine in the forebrain. Neuropharmacology.

[13] Zeng Y, Luo H, Gao Z, Zhu X, Shen Y, Li Y, Hu J, Yang J (2021). Reduction of prefrontal purinergic signaling is necessary for the analgesic effect of morphine. iScience.

[14] Zhang X, Lei B, Yuan Y, Zhang L, Hu L, Jin S, Kang B, Liao X, Sun W, Xu F (2020). Brain control of humoral immune responses amenable to behavioural modulation. Nature.

[15] Yuan Y, Wu W, Chen M, Cai F, Fan C, Shen W, Sun W, Hu J (2019). Reward inhibits paraventricular CRH neurons to relieve stress. Curr Biol.

[16] Liu Y, Zhang Y, Zheng X, Fang T, Yang X, Luo X, Guo A, Newell KA, Huang XF, Yu Y (2018). Galantamine improves cognition, hippocampal inflammation, and synaptic plasticity impairments induced by lipopolysaccharide in mice. J Neuroinflam.

[17] Huang X, Huang P, Huang L, Hu Z, Liu X, Shen J, Xi Y, Yang Y, Fu Y, Tao Q (2021). A visual circuit related to the nucleus reuniens for the spatial-memory-promoting effects of light treatment. Neuron.

[18] Taleb A, Zhou YP, Meng LT, Zhu MY, Zhang Q, Naveed M, Li LD, Wang P, Zhou QG, Meng F, Han F (2021). New application of an old drug proparacaine in treating epilepsy via liposomal hydrogel formulation. Pharmacol Res.

[19] Sun B, Ramberger M, O'Connor KC, Bashford-Rogers RJM, Irani SR (2020). The B cell immunobiology that underlies CNS autoantibody-mediated diseases. Nat Rev Neurol.

[20] Ni Y, Shen D, Zhang Y, Song Y, Gao Y, Zhou Q, He L, Yin D, Wang Y, Song F (2021). Expanding the clinical spectrum of anti-IgLON5 disease: a multicenter retrospective study. Eur J Neurol.

[21] Gaig C, Ercilla G, Daura X, Ezquerra M, Fernandez-Santiago R, Palou E, Sabater L, Hoftberger R, Heidbreder A, Hogl B (2019). HLA and microtubule-associated protein tau H1 haplotype associations in anti-IgLON5 disease. Neurol Neuroimmunol Neuroinflamm.

[22] Hansen N, Grunewald B, Weishaupt A, Colaco MN, Toyka KV, Sommer C, Geis C (2013). Human Stiff person syndrome IgG-containing high-titer anti-GAD65 autoantibodies induce motor dysfunction in rats. Exp Neurol.

[23] Geis C, Weishaupt A, Hallermann S, Grunewald B, Wessig C, Wultsch T, Reif A, Byts N, Beck M, Jablonka S (2010). Stiff person syndrome-associated autoantibodies to amphiphysin mediate reduced GABAergic inhibition. Brain.

[24] Haselmann H, Mannara F, Werner C, Planaguma J, Miguez-Cabello F, Schmidl L, Grunewald B, Petit-Pedrol M, Kirmse K, Classen J (2018). Human autoantibodies against the AMPA receptor subunit GluA2 induce receptor reorganization and memory dysfunction. Neuron.

[25] Haselmann H, Ropke L, Werner C, Kunze A, Geis C (2015). Interactions of human autoantibodies with hippocampal GABAergic synaptic transmission—analyzing antibody-induced effects ex vivo. Front Neurol.

[26] Colgin LL, Denninger T, Fyhn M, Hafting T, Bonnevie T, Jensen O, Moser MB, Moser EI (2009). Frequency of gamma oscillations routes flow of information in the hippocampus. Nature.

[27] Werner J, Jelcic I, Schwarz EI, Probst-Muller E, Nilsson J, Schwizer B, Bloch KE, Lutterotti A, Jung HH, Schreiner B (2021). Anti-IgLON5 disease: a new bulbar-onset motor neuron mimic syndrome. Neurol Neuroimmunol Neuroinflamm.

[28] Chen H, Wu J, Irani SR (2020). Distinctive magnetic resonance imaging findings in IgLON5 antibody disease. JAMA Neurol.

[29] Garcia-Serra A, Radosevic M, Pupak A, Brito V, Rios J, Aguilar E, Maudes E, Arino H, Spatola M, Mannara F (2021). Placental transfer of NMDAR antibodies causes reversible alterations in mice. Neurol Neuroimmunol Neuroinflamm.

[30] Carceles-Cordon M, Mannara F, Aguilar E, Castellanos A, Planaguma J, Dalmau J (2020). NMDAR antibodies alter dopamine receptors and cause psychotic behavior in mice. Ann Neurol.

[31] Ryding M, Gamre M, Nissen MS, Nilsson AC, Okarmus J, Poulsen AAE, Meyer M, Blaabjerg M (2021). Neurodegeneration induced by anti-IgLON5 antibodies studied in induced pluripotent stem cell-derived human neurons. Cells.

[32] Peng X, Hughes EG, Moscato EH, Parsons TD, Dalmau J, Balice-Gordon RJ (2015). Cellular plasticity induced by anti-alpha-amino-3-hydroxy-5-methyl-4-isoxazolepropionic acid (AMPA) receptor encephalitis antibodies. Ann Neurol.

[33] Jurek B, Chayka M, Kreye J, Lang K, Kraus L, Fidzinski P, Kornau HC, Dao LM, Wenke NK, Long M (2019). Human gestational *N*-methyl-d-aspartate receptor autoantibodies impair neonatal murine brain function. Ann Neurol.

[34] Zhou Q, Zhu X, Meng H, Zhang M, Chen S (2020). Anti-dipeptidyl-peptidase-like protein 6 encephalitis, a rare cause of reversible rapid progressive dementia and insomnia. J Neuroimmunol.

[35] Zhang XQ, Xu L, Ling Y, Hu LB, Huang J, Shen HW (2021). Diminished excitatory synaptic transmission correlates with impaired spatial working memory in neurodevelopmental rodent models of schizophrenia. Pharmacol Biochem Behav.

[36] Xu X, An L, Mi X, Zhang T (2013). Impairment of cognitive function and synaptic plasticity associated with alteration of information flow in theta and gamma oscillations in melamine-treated rats. PLoS ONE.

[37] Liu J, Chang L, Roselli F, Almeida OF, Gao X, Wang X, Yew DT, Wu Y (2010). Amyloid-beta induces caspase-dependent loss of PSD-95 and synaptophysin through NMDA receptors. J Alzheimers Dis.

[38] Coley AA, Gao WJ (2018). PSD95: a synaptic protein implicated in schizophrenia or autism?. Prog Neuropsychopharmacol Biol Psychiatry.

[39] Hu H, Gan J, Jonas P (2014). Interneurons. Fast-spiking, parvalbumin(+) GABAergic interneurons: from cellular design to microcircuit function. Science.

[40] Trimper JB, Stefanescu RA, Manns JR (2014). Recognition memory and theta-gamma interactions in the hippocampus. Hippocampus.

[41] Montgomery SM, Buzsáki G (2007). Gamma oscillations dynamically couple hippocampal CA3 and CA1 regions during memory task performance. Proc Natl Acad Sci USA.

[42] Ji MH, Lei L, Gao DP, Tong JH, Wang Y, Yang JJ (2020). Neural network disturbance in the medial prefrontal cortex might contribute to cognitive impairments induced by neuroinflammation. Brain Behav Immun.

[43] Hudson MR, Sokolenko E, O'Brien TJ, Jones NC (2020). NMDA receptors on parvalbumin-positive interneurons and pyramidal neurons both contribute to MK-801 induced gamma oscillatory disturbances: complex relationships with behaviour. Neurobiol Dis.

[44] Thierry AM, Gioanni Y, Dégénétais E, Glowinski J (2000). Hippocampo-prefrontal cortex pathway: anatomical and electrophysiological characteristics. Hippocampus.

[45] Benthem SD, Skelin I, Moseley SC, Stimmell AC, Dixon JR, Melilli AS, Molina L, McNaughton BL, Wilber AA (2020). Impaired hippocampal–cortical interactions during sleep in a mouse model of Alzheimer's disease. Curr Biol.

[46] Martorell AJ, Paulson AL, Suk HJ, Abdurrob F, Drummond GT, Guan W, Young JZ, Kim DN, Kritskiy O, Barker SJ (2019). Multi-sensory gamma stimulation ameliorates Alzheimer's-associated pathology and improves cognition. Cell.

[47] Liddelow SA, Guttenplan KA, Clarke LE, Bennett FC, Bohlen CJ, Schirmer L, Bennett ML, Munch AE, Chung WS, Peterson TC (2017). Neurotoxic reactive astrocytes are induced by activated microglia. Nature.

[48] Lian H, Litvinchuk A, Chiang AC, Aithmitti N, Jankowsky JL, Zheng H (2016). Astrocyte-microglia cross talk through complement activation modulates amyloid pathology in mouse models of Alzheimer's disease. J Neurosci.

[49] Cagnin A, Mariotto S, Fiorini M, Gaule M, Bonetto N, Tagliapietra M, Buratti E, Zanusso G, Ferrari S, Monaco S (2017). Microglial and neuronal TDP-43 pathology in anti-IgLON5-related tauopathy. J Alzheimers Dis.

[50] Strippel C, Heidbreder A, Schulte-Mecklenbeck A, Korn L, Warnecke T, Melzer N, Wiendl H, Pawlowski M, Gross CC, Kovac S. Increased intrathecal B and plasma cells in patients with anti-IgLON5 disease: a case series. Neurol Neuroimmunol Neuroinflamm 2022; 9. 10.1212/NXI.0000000000001137 PMC8759718 35031586

[51] Karis K, Eskla KL, Kaare M, Taht K, Tuusov J, Visnapuu T, Innos J, Jayaram M, Timmusk T, Weickert CS (2018). Altered expression profile of IgLON family of neural cell adhesion molecules in the dorsolateral prefrontal cortex of schizophrenic patients. Front Mol Neurosci.

[52] Yin D, Chen S, Liu J (2021). Sleep disturbances in autoimmune neurologic diseases: manifestation and pathophysiology. Front Neurosci.

[53] Mannara F, Radosevic M, Planaguma J, Soto D, Aguilar E, Garcia-Serra A, Maudes E, Pedreno M, Paul S, Doherty J (2020). Allosteric modulation of NMDA receptors prevents the antibody effects of patients with anti-NMDAR encephalitis. Brain.

