# Supplementary material for: Anti-IgLON5 antibodies cause progressive behavioral and neuropathological changes in mice
Source: J Neuroinflammation. 2022 Jun 11;19:140. doi: 10.1186/s12974-022-02520-z (PMC9188070; doi:10.1186/s12974-022-02520-z)
Supplement: Supplementary file 1 — Additional file 1: Supplemental materials for the methodology section. [file 12974_2022_2520_MOESM1_ESM.docx]

**Supplemental material**

**Legends to supplemental figures**

**Supplementary Figure 1: Clinical information of an anti-IgLON5 disease patient. Related to Figure 1. (A)** Representative images of cell-based assays showing anti-IgLON5 antibodies in the serum from the patient but not in the serum from a healthy control. Scale bar=20μm. **(B)** The cranial 18F-FDG PET-MR image of the patient upon first admission. The standardized uptake value ratios (SUVR) of the left thalamus, right thalamus, and left temporal lobe are 1.12, 0.97, and 0.91, respectively. The cranial 18F-FDG PET-MR of the patient upon the second admission. The SUVRs of the bilateral thalamus and left temporal lobe are lower than they are 18 months ago (left thalamus, 0.97; right thalamus, 0.82; left temporal lobe, 0.70). **(C)** Immunoprecipitation results show the presence of these antibodies in patient but their absence in control serum. **(D)** Timeline of symptoms.

**Supplementary Figure 2: Minor pathogenicity dose of anti-IgLON5 IgG and Metabolism in mice. Related to Figure 2. (A)** We diluted the patient's IgG by 10-fold and 100-fold with PBS respectively (0.23mg/ml and 0.023mg/ml). The novel object recognition index of 1/10 anti-IgLON5 IgG injected mice is lower than healthy control IgG-injected mice at Day 7 (n=6 per group, p=0.0419, one-way ANOVA with Tukey’s post-hoc test). **(B)** In the Y-maze, there is a reduced discrimination rate in 1/10 anti-IgLON5 IgG injected mice than the other two groups at Day 7 (Control-IgG vs. 1/10 anti-IgLON5 IgG: n=6 per group, p=0.001, one-way ANOVA with Tukey’s post-hoc test; 1/10 anti-IgLON5 IgG vs. 1/100 anti-IgLON5 IgG: n=6 per group, p=0.0097, one-way ANOVA with Tukey’s post-hoc test). **(C)** Body weights were tested every 2 days after final injection at the same time period and recorded again 30 days after the injection (Day 8: p=0.0193, unpaired t-test; Day 15: p=0.0406, unpaired t-test). **(D)** Rectal body temperature was measured every 2 days after final injection and recorded again 30 days after the injection. **(E)** Food intake were measured once every 2 days after final injection at the same time and recorded again 30 days after the injection (Day 13: p=0.0059, unpaired t-test). **(F)** Water intake were measured once every 2 days after final injection at the same time and recorded again 30 days after the injection (Day 13: p=0.0208, unpaired t-test). For (C)–(F), n=6 per group. *p < 0.05, **p < 0.01, ***p < 0.001 vs control IgG group.

**Supplementary Figure 3: The lower magnification views of Fig.4 E and G. Related to Figure 4.** **(A)** The lower magnification views of Fig.4 E and G have provided. In the representative pictures of NeuN expression in the hippocampus on day 7 (left), the black rectangles that appear at the corners of these images are areas that are not scanned by the confocal microscope. Because it is outside the target area, the analysis result is not affected. The white box area is the area we present in the results. Scale bars = 100µm.

**Supplementary Figure 4: Anti-IgLON5 antibodies reduce associated neurotransmitters. Related to Figure 6. (A)** Concentration of Glu in the hippocampus of control and anti-IgLON5 IgG group (n=3 per group, t=2.822, df=4, p=0.0477, unpaired t-test). **(B)** Concentration of GABA in the hippocampus of control and anti-IgLON5 IgG group. **(C)** Concentration of NE in the hippocampus of control and anti-IgLON5 IgG group. **(D)** Cultures treated with anti-IgLON5 IgG showed a decrease in PSD-95. Scale bar=25μm.

**Supplementary Figure 5: Anti-IgLON5 antibodies reduce cell surface synaptic proteins. Related to Figure 6. (A)** Immunoblot analysis of NMDAR1, PSD-95, and Synaptophysin expression in brains of anti-IgLON5 IgG and control-IgG treated mice. **(B)** Immunoblot analysis of NMDAR1, PSD-95, and Synaptophysin expression in cultures exposed to anti-IgLON5 IgG and control-IgG. **(C)** QRT-PCR results of related inflammatory markers and chemokines: TGF-β, IL-6, IL-1β, TNF-α, CXCL16, CXCR6, CCL5, CCL12, CXCL13 (TGF-β: n=3 per group, t=5.739, df=4, p=0.0046, unpaired t-test; CCL5: n=3 per group, t=18.49, df=4, p<0.0001, unpaired t-test; CXCL13: n=3 per group, t=3.707, df=4, p=0.0207, unpaired t-test). *p < 0.05, **p < 0.01, ***p < 0.001, ****p < 0.0001 vs control IgG group.

# **Materials and methods**

## Patient information

Detailed clinical information was obtained from patient and/or the patient's families or clinical records. Information regarding age of onset, disease progression, treatment and prognosis, and other clinical manifestations was collected. Detailed neurological examinations were carried out by neurologists. Antibody testing was performed using a cell-based assay to test for anti-IgLON5 antibodies by Kingmed Diagnostics Co., Ltd. A hybrid 18F-fluorodeoxyglucose (FDG) positron emission tomography (PET)/MRI scan was performed with a Biograph mMR system (Siemens, Erlangen, Germany) with a National Electrical Manufacturers Association (NEMA) PET resolution of 4.2 mm.

The patient was a 74-year-old woman. The main manifestations of her were cognitive impairment and anxiety, accompanied by mild sleep disorders. On neurological examination, her visual field, muscle strength, muscle tone and gait tests were normal. She had very high titers of IgLON5 IgG in the serum (1:1000) and cerebrospinal fluid (1:100) via immunofluorescence assay (Supplementary Figure 1A). They were mixed IgG4 (1:30) and IgG1 (1:100) subtypes, with IgG1 predominance. Other autoantibodies against neuronal surface antigens were negative. She carried the HLA DRB1*10:01- DQB1*05:01 haplotype, which is present in approximately 60-70% of patients with IgLON5 antibodies. Cranial ^18^F-fluorodeoxyglucose positron emission tomography magnetic resonance imaging (^18^F-FDG PET-MR) indicated mild hypometabolism in the left temporal lobe. The standardized uptake value ratios (SUVR) of the left thalamus, right thalamus and left temporal lobe was 1.12, 0.97, and 0.91, respectively. Follow-up cranial ^18^F-FDG PET-MR revealed that the SUVRs of the bilateral thalamus and left temporal lobe were lower than those 18 months ago (left thalamus, 0.97; right thalamus, 0.82; left temporal lobe, 0.70).

## Behavioral testing

**Novel object recognition**

The novel object recognition test was used to evaluate long-term memory [1]. Mice were placed in the apparatus to become familiar with the testing environment for 10 min for two successive days before object exposure. On the day of testing, two objects were placed in the back left and right corners of the apparatus. Mice were placed at the midpoint of the wall opposite the objects and were then exposed to the objects for 10 min before being returned to the home cage. Three hours later, one of the two objects was replaced by another novel object and mice were exposed to the two objects for another 10 min. Object exploration was defined as each instance in which a mouse’s nose touched the object or was oriented toward it and came within 2 cm [2]. Movements were recorded with a video camera and analyzed by another member of our group who was blinded to mice treatment. A discrimination index was calculated as the difference of the time spent exploring the novel and the time spent exploring the familiar object divided by the total time exploring both objects. The higher the discrimination index, the better the memory retention ability of familiar objects.

**Y-maze**

We tested short-term spatial working memory by recording spontaneous alternation behavior in the Y-maze. Mice were placed at the end of one arm and allowed to explore all three arms freely for 5 min. The computer recording system was started to track the mice. Three consecutive choices of all three arms were regarded as a correct alternation. The percentage of spontaneous alternation was calculated by dividing the total number of alternations by the total number of choices minus 2 [3]. Movements were recorded with a video camera and analyzed by another member of our group who was blinded to mice treatment.

**Elevated plus maze (EPM)**

The elevated plus maze (EPM) test is an experimental method for evaluating the exploration and anxiety responses in rodents. This maze consisted of two open arms facing in opposite directions and two closed arms facing in opposite directions that were 50 cm above the floor (all arms were 7 cm wide and 27 cm long). As detailed previously [4], at the start of the test, mice were placed in the center of the maze facing an open arm and allowed to roam freely for 5 min, during which a video tracking device recorded their movement and behaviors. Both the time spent in open arms and the percentage of time spent in open arms were measured as exploratory behaviors of interest, with an arm entry being recorded when a mouse entered a given maze arm with all four paws.

**Open field test**

Mice were placed in the center zone of a 38*38*38 cm open field chamber in a room with dim light for 10 min. A video camera positioned directly above the chamber was used to record the movement of each test mouse. An automated video-tracking system was controlled by MATLAB R2018b. Therefore, the total distance traveled during the session was tracked and further analyzed.

**Three-chamber social interaction test**

The three-chamber testing apparatus consisted of a center compartment (20 cm × 25 cm × 30cm) and two end compartments, which were of equal size (20 cm × 25 cm× 30cm). Inverted custom-made wire cups (diameter 8 cm) were placed in each side of the end compartments during testing sessions (discussed below) and housed the stranger mouse. All stranger mice were males of the same age and previously habituated to the plastic cage for 30 min during the previous day. The apparatus and wire cups were thoroughly cleaned with 75% ethanol between sessions and after each test mouse.

In the first session, a test mouse was placed in the middle compartment and allowed to habituate to the apparatus for 10 min. Then, the mouse was gently guided to its cage, and a stranger mouse was placed in an inverted wire cup in the side designated as the social compartment, and an empty inverted wire cup was placed in the side designated as the nonsocial compartment. In the second phase (sociability), the subject mouse was allowed to explore the new environment freely for 10 min. Behavioral tests were recorded by a video camera, and the amount of time test mice spent in each compartment, the amount of time they explored (sniffing) within a 2 cm vicinity of the inverted cups, and their transitions between compartments were measured[5].

**Rotarod test**

Motion coordination ability was evaluated by rotarod test[6]. The mice were placed on a rotating rod, facing away from the direction of rotation. The rotarod was initially set with a speed of 4 rpm for the initial 10 seconds after which an acceleration of 20 rpm/minute was applied. Time to fall was recorded. If the mouse had fallen off during the initial 10 seconds it would have another try, to a maximum of 3 trials.

**Tail suspension (TST)**

Mice were suspended by binding their tail with an adhesive tape that was fastened to a rail 50 cm from the ground. The joint height was high enough to ensure that the mice could not make other contact or climb during the test. A six-minute video recording began immediately after the mice were taped. The immobility duration in the last 4 min of the experiment was counted by another member of our group who was blinded to mice treatment[7].

**Food intake and metabolism**

The body weight of the mice was measured once before surgery and recorded as initial weight. Body weights were tested every 2 days after final injection at the same time period and recorded again 30 days after the injection. Food and water intake were measured once every 2 days after final injection at the same time and recorded again 30 days after the injection. Rectal body temperature was measured every 2 days after final injection, as described previously.

**Histology**

Methods of cell quantitative analysis were referred to previous literature[8]. Confocal images were taken across the z-plane spanning the entire hippocampus in all cases using a Nikon CSU 2 Camera confocal microscope. Exposure time and laser intensity settings were held constant during image collection. Each mouse selected 3 brain slices, and the CA1 region on one side of each brain slice was selected as a section. Finally, each mouse obtained a total of 3 images and calculated their average density. Twelve stacks were taken every 1.5 μm z-plane for the hippocampus. For the neuronal density, cells were counted in every sixth image in the same stack. Cells/µm2 reported reflect mean density values across sampled sections. Images were analyzed on ImageJ and Qupath. We set the same threshold value for all images (including the same nuclear parameters, cell parameters and general parameters). Due to technical limitations, Qupath software could not save ROIs, so we chose similar ROIs as much as possible, and controlled the area of each group of CA1 region within 50,000-60,000 μm2 to reduce error. After Qupath analysis, cells above the threshold value were identified as positive cells.

For astrocyte and microglial densities, images were z-projected and the density calculated as number of cells per unit of area using ImageJ and Qupath. The z-step/interval was 1.5 μm within a 40 μm depth. An average density was obtained (cells/mm2) for each area.

For astrocyte and microglial densities, images were z-projected and cells counted and the density calculated as number of cells per volume of area using FiJi and Qupath.

For the beauty of the picture, the green Iba-1 staining is digitally made red with the help of ImageJ.

## In vitro electrophysiological recordings

Bilateral injections of purified and concentrated AAV-CaMKIIα-ChR2-mCherry virus (2×10^12^ vector genomes/mL, Neuron Biotech Company, China) were stereotaxically performed in 5-week-old male C57BL/6 J mice. Each side of CA1(AP, -1.4 mm; ML, ±1.0 mm; DV, −1.7 mm) was injected with 300nL AAV for 10 min followed by an additional 10 min to allow the diffusion of viral particles away from the injection site. The virus was allowed to express for a minimum of 2 weeks in order to allow time for sufficient opsin accumulation in the axons. Mice were then treated with anti-IgLON5 IgG or control IgG for 7 days as described above. Mice were anesthetized with isoflurane and perfused immediately with ice-cold NMDG artificial cerebrospinal fluid (ACSF). The pipettes were filled with ACSF solution containing 133 mM potassium gluconate, 18 mM NaCl, 0.6 mM EGTA, 10 mM HEPES, 2 mM Mg·ATP, and 0.3 mM Na3·GTP (pH:7.2, 280 mOsm). After the whole-cell record was formed, the neurons were held at -70 mV under a voltage-clamp mode to record spontaneous excitatory postsynaptic currents (sEPSCs), the sEPSCs were recorded for 5min. For the action potentials that evoked by current injections, a current-step protocol (from -20 to 200 pA, with 20 pA increments) was run and repeated. To observe PPF, two synaptic responses were evoked by a pair of optical stimulating pulses given at short intervals (50 ms) at 0.1 Hz [18]. Recordings with Rs > 30 MΩ were not statistically analyzed. All recordings were acquired using a Multiclamp 700B amplifier and signals were low-pass filtered at 2 kHz and digitized at 10 kHz (DigiData 1550, Molecular Devices).

The physiological data were analyzed using Clampfit 10 software (Molecular Devices) and Mini Analysis Program (Synaptosoft). sEPSCs were analyzed by Mini Analysis Program with an amplitude threshold of 8 pA. Summary data are presented as mean ± SEM. *p < 0.05 was used to designate an effect as significant.

## Western blot analysis

Equal amounts of protein were separated by 10% SDS-polyacrylamide gel electrophoresis and transferred onto nitrocellulose membranes. Membranes were blocked via 5% skim milk powder in Tris-buffered saline including 0.05% (v/v) Tween 20 (TBST) for 2 h at 25 °C and then incubated overnight with the primary antibodies to NMDAR1(1:1000, #32-0500, Invitrogen), synaptophysin (1:1000, ab32127, abcam), PSD-95 (1:1000, ab238135, abcam) and β-actin (1:1000, TA-09, ZSGB-BIO). Membranes were washed thrice with TBST over 15 min and incubated with secondary antibodies (ZSGB-BIO, Beijing, China) in 5% skim milk powder in TBST. The membranes were exposed to BCIP/NBT alkaline phosphatase color developing reagent (Beyotime Institute of Biotechnology, Shanghai, China) for 15 min. Bands corresponding to the proteins of interest were scanned and band density analyzed using the Quantity One automatic imaging analysis system (Bio-Rad).

## Immunoprecipitation

Serum antibody-bound magnetic beads were prepared by incubation 15 μL Pierce Protein A/G Magnetic Beads (88802, Thermo Scientific™) in 500 μL blocking buffer (TBST containing 5% goat serum) supplemented with 10 μL healthy control or anti-IgLON5 positive patient serum at RT for 4 hours followed by washing with 1 mL TBST 3 times. IgLON5 lysates were prepared from HEK293T cells transfected with pcDNA3.1-IgLON5. In brief, 20 million transfected cells were collected by 1 mL lysis buffer (TBS, pH 7.4, containing 1% TritonX-100 supplemented with protease inhibitor cocktail), vortexed for 1 min and incubate on ice for 10 min. Insoluble fraction were pelleted by centrifuge at 12000 rpm for 5 min and the supernatant was collected for IP. Fifty microliter of the supernatant was used as input and 400 μL were used for IP by incubation with control or patient serum antibody-bound magnetic beads at 4 ℃ overnight. The magnetic beads were washed with TBST 5 times followed by elution with 1X Laemmli buffer. Western blot detecting IgLON5 were performed by a standard protocol using a commercial antibody (1:1000, #ab122763, Abcam).

## UHPLC/MS

The ultrahigh performance liquid chromatography-mass spectrometry (UHPLC-MS) method was established to quantify three neurotransmitters in the hippocampus: Glu (Glutamic acid), GABA (γ-aminobutyric acid) and NE (Norepinephrine) [9, 10]. We collected hippocampal samples from the test mice under tribromoethanol. The samples were prepared on ice and weighed. The sample treatment and data analysis were carried out by Wuhan Servicebio technology CO., LTD.

## QRT-PCR (quantitative Real time PCR)

Reverse transcriptase and quantitative Real time PCR analyses was used to measure the expression intensity of different genes. Total RNA was extracted from hippocampus tissue using TRIzol reagent (Servicebio). The purity and quantity of RNA was detected using NanoDrop 2000 spectrophotometer (Thermo Fisher Scientific, Waltham, MA, USA). The RNA was reversely transcribed to complementary DNA using PrimeScript™ RT Reagent Kit (Takara, Tokyo, Japan). QRT-PCR was carried out by Wuhan Servicebio technology CO., LTD according to the instruction. QRT-PCR was performed using the following steps: pre-denaturation at 95°C for 10 min, then 40 cycles of denaturation at 95°C for 15 s and annealing at 60°C for 60 s. From 60℃ to 95℃, fluorescence signals were detected every rise of 0.3℃. The results were analyzed using the ∆∆CT (cycle threshold) method for quantification. GADPH was used as an internal control.

Details of the primers are summarized in Table 1.

Table1 Primer sequences for real-time quantitative polymerase chain reaction (qRT-PCR).

| Gene |  |  | Sequence (5'-3') |
| --- | --- | --- | --- |
| GAPDH | Forward primer | | CCTCGTCCCGTAGACAAAATG |
|  | Reverse primer | | TGAGGTCAATGAAGGGGTCGT |
| TGF-β1(r3) | Forward primer | | AACAATTCCTGGCGTTACCTT |
|  | Reverse primer | | TCGAAAGCCCTGTATTCCGTCT |
| IL-6 | Forward primer | | TTCTTGGGACTGATGCTGGTG |
|  | Reverse primer | | CACAACTCTTTTCTCATTTCCACGA |
| TNF-α(rz) | Forward primer | | TGGAACTGGCAGAAGAGGCAC |
|  | Reverse primer | | AGGGTCTGGGCCATAGAACTGA |
| IL-1β | Forward primer | | TGCCACCTTTTGACAGTGATG |
|  | Reverse primer | | CATCTCGGAGCCTGTAGTGC |
| CCL5 | Forward primer | | TGCCCACGTCAAGGAGTATTT |
|  | Reverse primer | | GATGTATTCTTGAACCCACTTCTTC |
| CCL12 | Forward primer | | GATTTCCACACTTCTATGCCTCC |
|  | Reverse primer | | GGCTGCTTGTGATTCTCCTGT |
| CXCL13(rz) | Forward primer | | AATGGCTGCCCCAAAACTGA |
|  | Reverse primer | | GGGAGTTGAAGACAGACTTTTGC |
| CXCL16 | Forward primer | | GCAAAGAGTGTGGAACTGGTCA |
|  | Reverse primer | | GCTGTCTGTCATCTTGCTGTTTC |
| CXCR6 | Forward primer | | TGCTTGCTCATTTGGGTGGT |
|  | Reverse primer | | CATAGTGAGCAATGGCAGGAAG |

## Supplemental Reference

1. Antunes M, Biala G: **The novel object recognition memory: neurobiology, test procedure, and its modifications.** *Cogn Process* 2012, **13:**93-110.

2. Bevins RA, Besheer J: **Object recognition in rats and mice: a one-trial non-matching-to-sample learning task to study 'recognition memory'.** *Nat Protoc* 2006, **1:**1306-1311.

3. Cao ZP, Dai D, Wei PJ, Han YY, Guan YQ, Li HH, Liu WX, Xiao P, Li CH: **Effects of cordycepin on spontaneous alternation behavior and adenosine receptors expression in hippocampus.** *Physiol Behav* 2018, **184:**135-142.

4. Zhang JY, Liu TH, He Y, Pan HQ, Zhang WH, Yin XP, Tian XL, Li BM, Wang XD, Holmes A, et al: **Chronic Stress Remodels Synapses in an Amygdala Circuit-Specific Manner.** *Biol Psychiatry* 2019, **85:**189-201.

5. Wang X, McCoy PA, Rodriguiz RM, Pan Y, Je HS, Roberts AC, Kim CJ, Berrios J, Colvin JS, Bousquet-Moore D, et al: **Synaptic dysfunction and abnormal behaviors in mice lacking major isoforms of Shank3.** *Hum Mol Genet* 2011, **20:**3093-3108.

6. Deacon RM: **Measuring motor coordination in mice.** *J Vis Exp* 2013**:**e2609.

7. Can A, Dao DT, Terrillion CE, Piantadosi SC, Bhat S, Gould TD: **The tail suspension test.** *J Vis Exp* 2012**:**e3769.

8. Giannoccaro MP, Menassa DA, Jacobson L, Coutinho E, Prota G, Lang B, Leite MI, Cerundolo V, Liguori R, Vincent A: **Behaviour and neuropathology in mice injected with human contactin-associated protein 2 antibodies.** *Brain* 2019, **142:**2000-2012.

9. Tchoumtchoua J, Halabalaki M, Gikas E, Tsarbopoulos A, Fotaki N, Liu L, Nam S, Jove R, Skaltsounis LA: **Preliminary pharmacokinetic study of the anticancer 6BIO in mice using an UHPLC-MS/MS approach.** *J Pharm Biomed Anal* 2019, **164:**317-325.

10. Wang P, Gao X, Zhao F, Gao Y, Wang K, Tian JS, Li Z, Qin XM: **Study of the Neurotransmitter Changes Adjusted by Circadian Rhythm in Depression Based on Liver Transcriptomics and Correlation Analysis.** *ACS Chem Neurosci* 2021, **12:**2151-2166.
