# Supplementary figures and images for: Anti-IgLON5 antibodies cause progressive behavioral and neuropathological changes in mice
Source: J Neuroinflammation. 2022 Jun 11;19:140. doi: 10.1186/s12974-022-02520-z (PMC9188070; doi:10.1186/s12974-022-02520-z)

Supplementary Figure 1

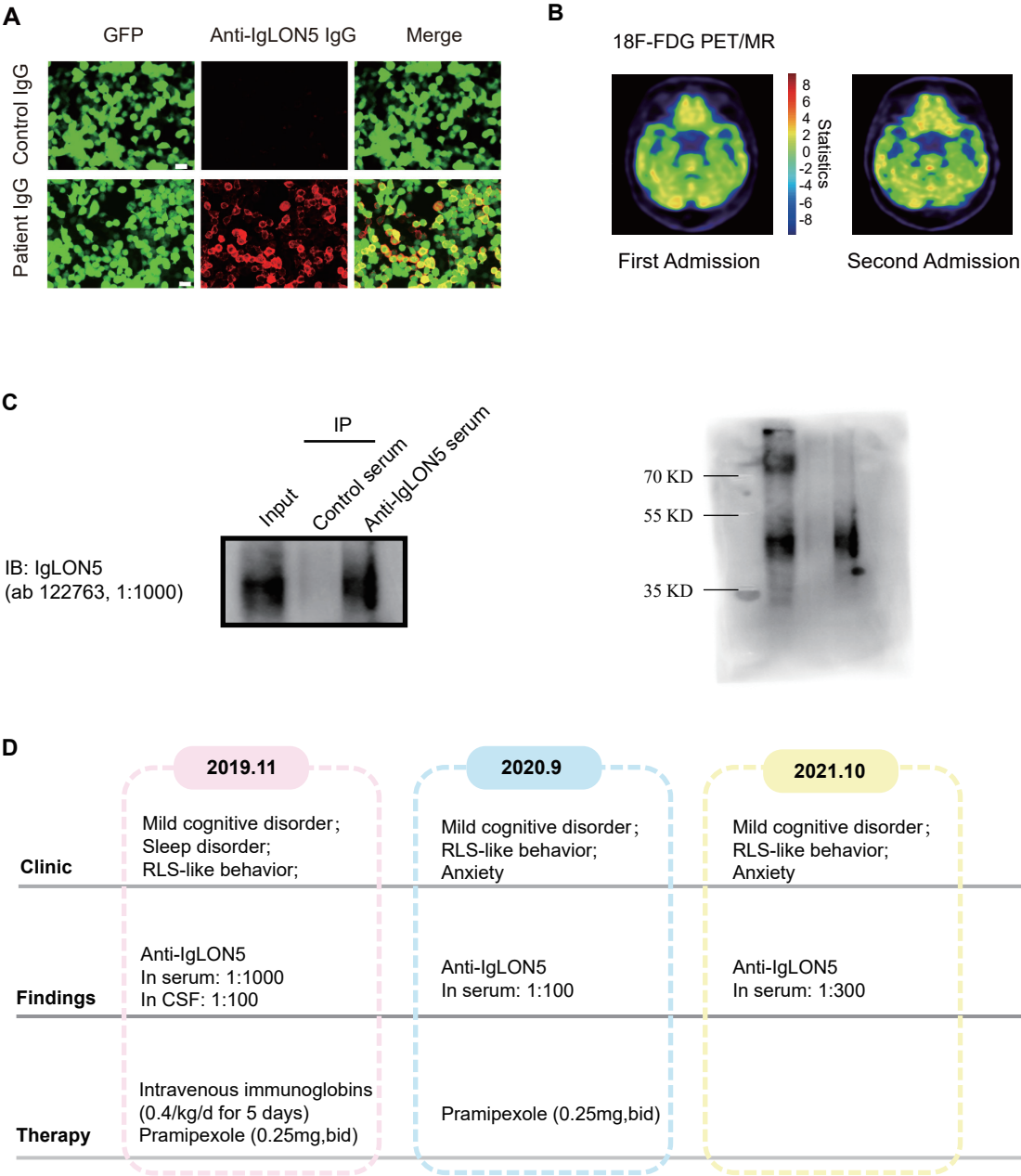

Supplement: Supplementary file 2 — Additional file 2: Fig. S1. Clinical information of an anti-IgLON5 disease patient. Related to Figure 1. (A) Representative images of cell-based assays showing anti-IgLON5 antibodies in the serum from the patient but not in the serum from a healthy control. Scale bar=20μm. (B) The cranial 18F-FDG PET-MR image of the patient upon first admission. The standardized uptake value ratios (SUVR) of the left thalamus, right thalamus, and left temporal lobe are 1.12, 0.97, and 0.91, respectively. The cranial 18F-FDG PET-MR of the patient upon the second admission. The SUVRs of the bilateral thalamus and left temporal lobe are lower than they are 18 months ago (left thalamus, 0.97; right thalamus, 0.82; left temporal lobe, 0.70). (C) Immunoprecipitation results show the presence of these antibodies in patient but their absence in control serum. (D) Timeline of symptoms. [file 12974_2022_2520_MOESM2_ESM.pdf]

Supplementary Figure 2

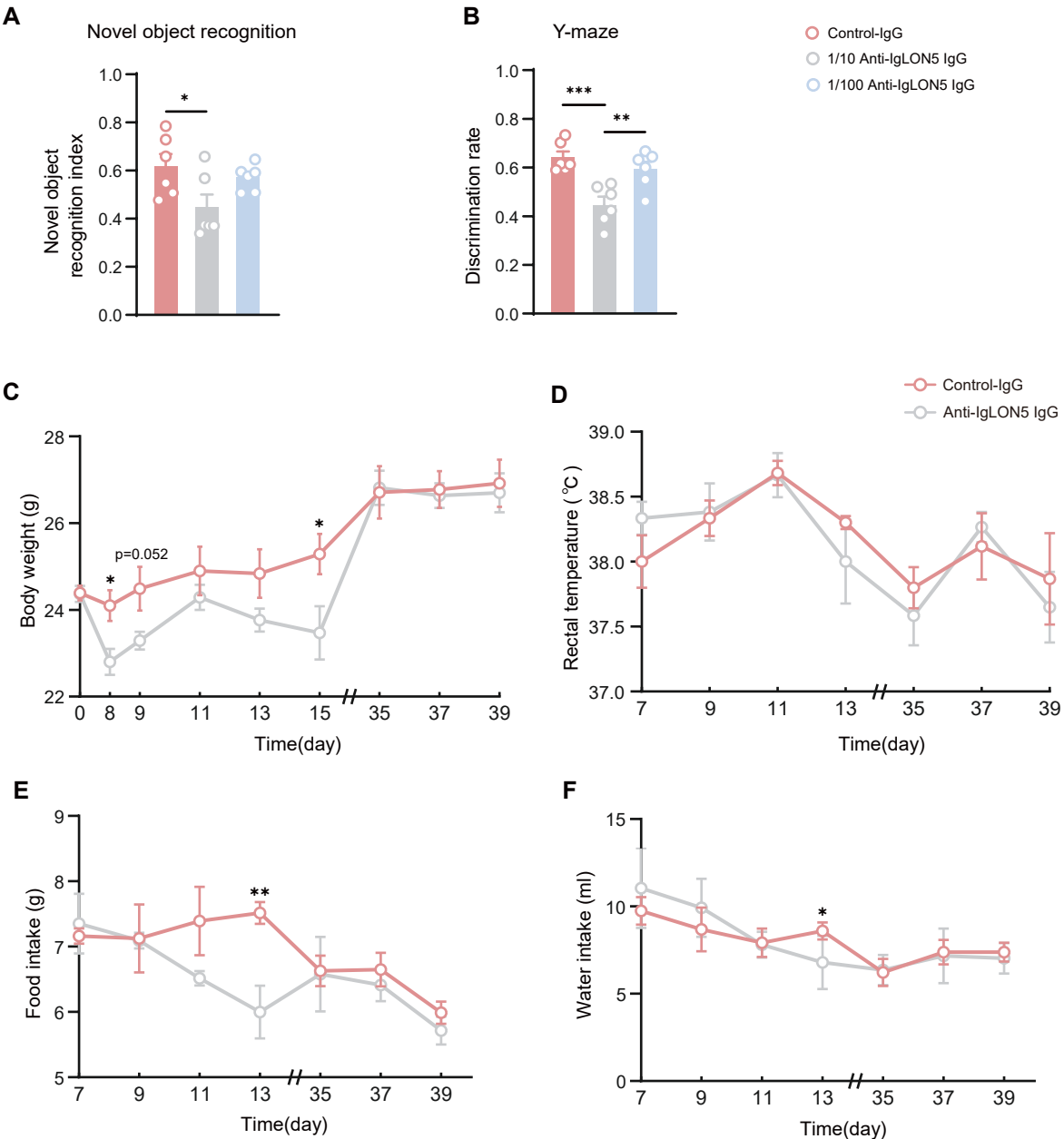

Supplement: Supplementary file 3 — Additional file 3: Fig. S2. Minor pathogenicity dose of anti-IgLON5 IgG and Metabolism in mice. Related to Figure 2. (A) We diluted the patient's IgG by 10-fold and 100-fold with PBS respectively (0.23mg/ml and 0.023mg/ml). The novel object recognition index of 1/10 anti-IgLON5 IgG injected mice is lower than healthy control IgG-injected mice at Day 7 (n=6 per group, p=0.0419, one-way ANOVA with Tukey’s post-hoc test). (B) In the Y-maze, there is a reduced discrimination rate in 1/10 anti-IgLON5 IgG injected mice than the other two groups at Day 7 (Control-IgG vs. 1/10 anti-IgLON5 IgG: n=6 per group, p=0.001, one-way ANOVA with Tukey’s post-hoc test; 1/10 anti-IgLON5 IgG vs. 1/100 anti-IgLON5 IgG: n=6 per group, p=0.0097, one-way ANOVA with Tukey’s post-hoc test). (C) Body weights were tested every 2 days after final injection at the same time period and recorded again 30 days after the injection (Day 8: p=0.0193, unpaired t-test; Day 15: p=0.0406, unpaired t-test). (D) Rectal body temperature was measured every 2 days after final injection and recorded again 30 days after the injection. (E) Food intake were measured once every 2 days after final injection at the same time and recorded again 30 days after the injection (Day 13: p=0.0059, unpaired t-test). (F) Water intake were measured once every 2 days after final injection at the same time and recorded again 30 days after the injection (Day 13: p=0.0208, unpaired t-test). For (C)–(F), n=6 per group. *p < 0.05, **p < 0.01, ***p < 0.001 vs control IgG group [file 12974_2022_2520_MOESM3_ESM.pdf]

## Supplementary Figure 3

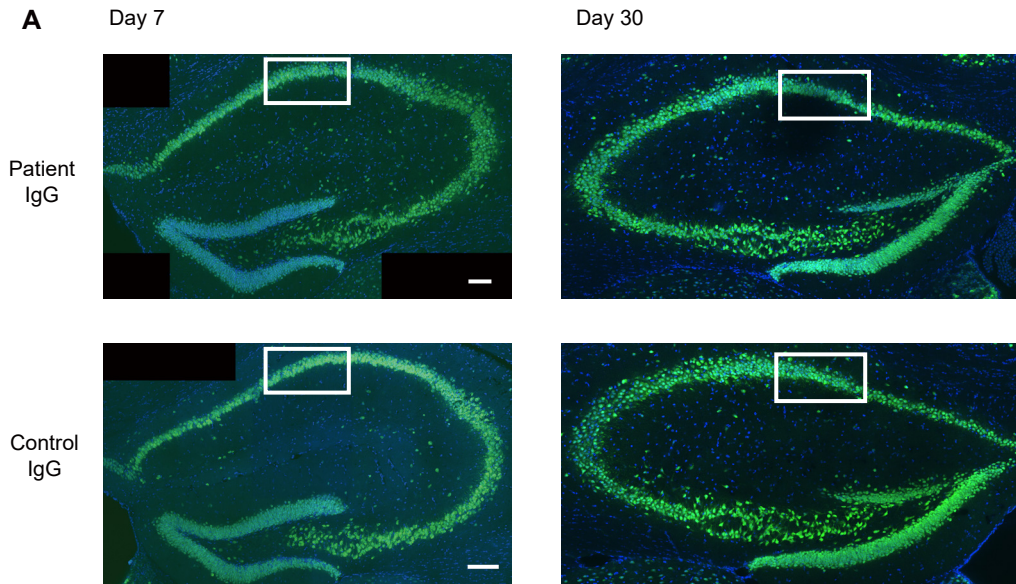

Supplement: Supplementary file 4 — Additional file 4: Fig. S3. The lower magnification views of Fig.4 E and G. Related to Figure 4. (A) The lower magnification views of Fig.4 E and G have provided. In the representative pictures of NeuN expression in the hippocampus on day 7 (left), the black rectangles that appear at the corners of these images are areas that are not scanned by the confocal microscope. Because it is outside the target area, the analysis result is not affected. The white box area is the area we present in the results. Scale bars = 100µm. [file 12974_2022_2520_MOESM4_ESM.pdf]

Supplementary Figure 4

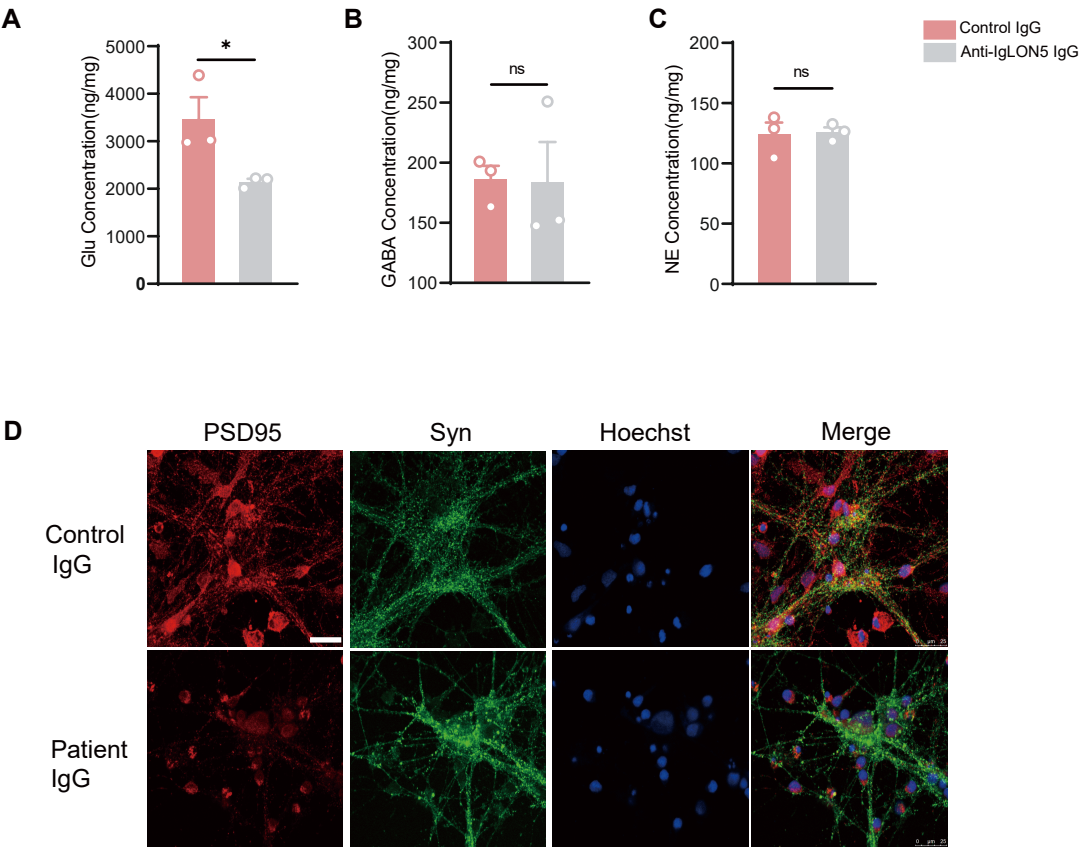

Supplement: Supplementary file 5 — Additional file 5: Fig. S4. Anti-IgLON5 antibodies reduce associated neurotransmitters. Related to Figure 6. (A) Concentration of Glu in the hippocampus of control and anti-IgLON5 IgG group (n=3 per group, t=2.822, df=4, p=0.0477, unpaired t-test). (B) Concentration of GABA in the hippocampus of control and anti-IgLON5 IgG group. (C) Concentration of NE in the hippocampus of control and anti-IgLON5 IgG group. (D) Cultures treated with anti-IgLON5 IgG showed a decrease in PSD-95. Scale bar=25μm. [file 12974_2022_2520_MOESM5_ESM.pdf]

Supplementary Figure 5

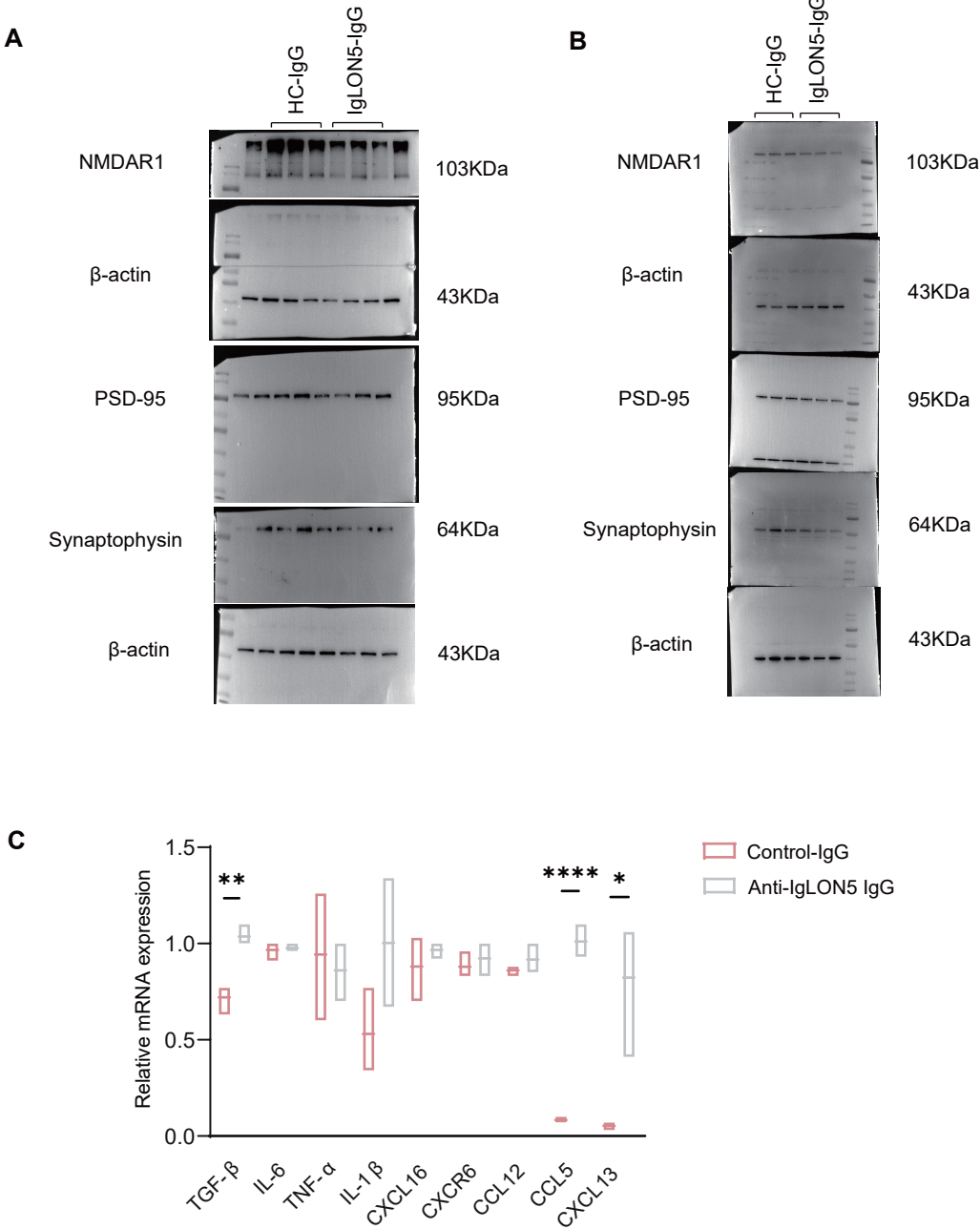

Supplement: Supplementary file 6 — Additional file 6: Fig. S5. Anti-IgLON5 antibodies reduce cell surface synaptic proteins. Related to Figure 6. (A) Immunoblot analysis of NMDAR1, PSD-95, and Synaptophysin expression in brains of anti-IgLON5 IgG and control-IgG treated mice. (B) Immunoblot analysis of NMDAR1, PSD-95, and Synaptophysin expression in cultures exposed to anti-IgLON5 IgG and control-IgG. (C) QRT-PCR results of related inflammatory markers and chemokines: TGF-β, IL-6, IL-1β, TNF-α, CXCL16, CXCR6, CCL5, CCL12, CXCL13 (TGF-β: n=3 per group, t=5.739, df=4, p=0.0046, unpaired t-test; CCL5: n=3 per group, t=18.49, df=4, p<0.0001, unpaired t-test; CXCL13: n=3 per group, t=3.707, df=4, p=0.0207, unpaired t-test). *p < 0.05, **p < 0.01, ***p < 0.001, ****p < 0.0001 vs control IgG group. [file 12974_2022_2520_MOESM6_ESM.pdf]
